# Spatioseasonal Variability and Correlations of Particle-Bound Organophosphate Esters in Providence, Rhode Island

**DOI:** 10.1021/acsomega.5c07462

**Published:** 2025-12-11

**Authors:** Annie Gathof, Leart Jahaj, Savannah Patalano, Adelaide E. Clark

**Affiliations:** Department of Chemistry and Biochemistry, 6753 Providence College, One Cunningham Square, Providence, Rhode Island 02915, United States

## Abstract

The concentrations of organophosphate esters (OPEs) in atmospheric particulate matter (PM) collected every 6 days at two sites across Providence, Rhode Island (Providence College, PC, and the Port of Providence, Port) are reported for the first time. Of the 31 OPEs in the target analyte list, 23 were detected in either total suspended particulate (TSP) and PM less than 2.5 μm in aerodynamic diameter (PM_2.5_), including six “novel” OPEs never before reported in the US. Notably, this study represents the longest, most comprehensive report of OPEs in PM_2.5_ in the US. Median total OPE concentrations (∑_23_OPE) were similar between sites (PC TSP: 600 pg m^–3^; PC PM_2.5_: 751 pg m^–3^; Port TSP: 689 pg m^–3^; Port PM_2.5_: 889 pg m^–3^), though the sites proved statistically different when compared by size fraction (*p* < 0.05), likely due to the variety of sources contributing to ∑_23_OPE. Tris­[(2*R*)-1-chloro-2-propyl] phosphate (TCPP) was the most prominent OPE in samples, with chlorinated OPEs dominating sample profiles regardless of site and size fraction. Select OPEs were significantly (*p* < 0.05) correlated with each other, suggesting similar sources in the area, though several deviations examined suggest source differences despite sites being 6.5 km apart. Meteorological parameters were also examined, with temperature proving an important factor for OPEs in both TSP and PM_2.5_, but relative humidity only affecting PM_2.5_ samples. Seasonal differences were also noted, with autumn and summer ∑_23_OPE concentrations significantly higher than winter and spring. Daily intensives during autumn and spring also showed high day-to-day variability in OPE concentrations, with summer night samples providing some of the highest OPE concentrations observed in the study. It is possible that traditional 1-in-6 or 1-in-12-day sampling schemes with samplers in one location in a city are not adequately characterizing OPEs in the atmosphere and more intensive strategies are needed for the best understanding of OPE ambient concentrations to better characterize their effects on human health in urban areas.

## Graphical abstract

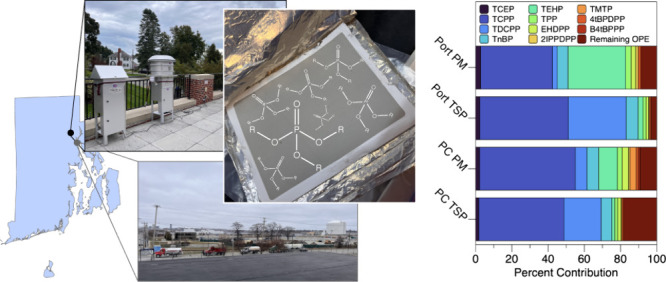

## Introduction

Air pollution and its associated health effects continue to be a major source of concern throughout the world. Atmospheric particulate matter (PM) remains an effective medium for the sorption of organic chemicals.^1^ Due to this sorption, the health effects associated with organic chemicals, such as the “reemerging” organophosphate esters (OPEs), must be considered with the hazards of air pollution.^2,3^

With more than 1000 tons produced annually,^4^ OPEs are classified as “high-production-volume” chemicals.^5^ Though they have a wide variety of uses in consumer products as plasticizers and solvents,^6–15^ they have been regarded as “ideal substitutes” for brominated flame retardants (BFRs).^7^ To that end, OPEs are “increasingly dwarfing” other replacement flame retardants (FRs)^16^ with an estimated growth in the market of 6.9% between 2018 and 2023 ^17^ and an estimated global consumption of over 2.6 million tons in 2018 (a 4-fold increase in 5 years after only doubling in the decade prior).^18,19^ Studies have confirmed this dominance, with OPE profiles 2–5 orders of magnitude higher than other FRs in a study of 20 global megacities.^20^

OPEs were first detected in the environment over 50 years ago, but these studies were mostly abandoned due to conclusions that OPEs were easily degradable in the environment.^21^ Since then, high concentrations of OPEs have been found ubiquitously in the environment, including in the Arctic where concentrations have increased in the past decade, suggesting long-range atmospheric transport.^5,22–25^ As this accumulation in the environment has occurred, questions have been raised about the risks they pose to human health.

Worldwide, high variability in OPE concentrations has been observed with concentrations as low as 35 pg m^–3^ (in the Atlantic and Arctic Oceans)^26^ to as high as 156,000 pg m^–3^ (in urban Guangzhou, China).^27^ Despite this, OPEs remain understudied in PM, with only five studies in the United States published since 2014.^28–32^ Data are even more limited for the respirable PM_2.5_ size fraction (PM 2.5 μm or less in aerodynamic diameter), with only one out of the five studies since 2014 looking at PM_2.5_ in the US^29^ and three available from China.^33–35^ With ever-increasing concerns about air pollution and climate change in the country, there is a strong basis to conduct a detailed analysis of OPEs in the US, especially in areas of high population (where consumer products containing OPEs will be prevalent) not previously studied.

The city of Providence, Rhode Island is one of the oldest industrialized cities in the country and is home to one of only two deep-water ports in New England.^36,37^ In addition to international trade, industry, and transportation, the city is also a dense urban environment. In the 2020 census, the city had a population of 190,978 and an estimated population density of 10,373.5 per square mile,^38^ the third most dense in New England (Cambridge, MA: 18,250.7; Boston, MA: 13,976.7)^39,40^ and higher than the city of Los Angeles (8304.2).^41^ Despite its population density, levels of OPEs in Providence (and greater New England) remain unstudied.

This research aims to expand the understanding of the ambient concentrations of the OPEs in the urban US, while adding to the understanding of spatioseasonal trends and trends across size fractions, which are not well-characterized. Specifically, the goals of this research were to (1) provide a comprehensive year-long study of OPEs in the US for the first time in 5 years, (2) examine spatial and temporal trends in OPEs throughout the year as they relate to each other and other meteorological parameters, and (3) examine trends in size fractions related to OPEs.

## Results and Discussion

A total of 23 OPEs were detected in at least one sample (Table S1), and 18 OPEs were common across sites and size fractions over the study period. At Providence College (PC, an urban site), 11 and 9 OPEs were detected in over 50% of the TSP and PM_2.5_ samples, respectively. At the Port of Providence (Port, an industrial site), 9 OPEs were detected in over 50% of the TSP and PM_2.5_ samples. Median and maximum ambient concentrations and detection frequencies for OPEs detected in over 50% of samples for each site and size fraction (and structural summations) are summarized in Table 1. Average surrogate recoveries are given for each site and size fraction in the Supporting Information (Table S2).

**Table 1:** Median (Med) and Maximum (Max) Ambient Concentrations (pg m^–3^) and Detection Frequencies (DF, %) of OPEs Detected in More Than 50% of Samples, by Size Fraction and Sampling Site^a^

	Providence College						Port of Providence					
	PM_2.5_ (*n* = 63)			TSP (*n* = 66)			PM_2.5_ (*n* = 26)			TSP (*n* = 23)		
	Med	Max	DF	Med	Max	DF	Med	Max	DF	Med	Max	DF
TCEP	13	170	70	13	79	82	19	64	69	19	49	91
TCPP	353	3440	97	320	2410	100	422	3390	100	273	1450	100
TDCPP	154	1540	100	39	142	86	275	2400	92	<MDL	55	78
∑Cl-OPE	528	5160	100	388	2630	100	698	5860	100	285	1480	100
TnBP	45	462	90	39	322	95	57	434	88	41	211	91
TEHP	<MDL	174	79	63	242	98	<MDL	56	96	220	327	100
∑Alkyl-OPE	54	568	98	127	416	100	75	455	100	254	379	100
TPP	<MDL	400	95	16	139	92	<MDL	114	77	21	95	91
EHDPP	<MDL	185	97	21	218	97	<MDL	84	92	<MDL	48	91
2IPPDPP	<MDL	165	59	<MDL	27	59	<MDL	28	59	<MDL	<MDL	96
TMTP				<MDL	91	64						
4tBPDPP	<MDL	53	83	<MDL	37	85	<MDL	24	65	<MDL	32	78
B4tBPPP				<MDL	20	53						
∑Aryl-OPE	52	1080	100	64	530	100	51	276	100	78	163	100
∑_23_OPE	751	6800	100	600	3220	100	889	6270	100	689	1950	100

a <MDL indicates that more than half of the detected ambient concentrations were less than the method detection limits.

The OPE target analyte list has expanded in recent years to include “novel” OPEs. 2-Isopropylphenyl diphenyl phosphate (2IPPDPP), bis­(4-*tert*-butylphenyl) phenyl phosphate (B4tBPPP), and 4-*tert*-butylphenyl diphenyl phosphate (4tBPDPP) were detected in more than 50% of samples from at least one site in this study. 2IPPDPP, a component of Firemaster (FM) 550,^42^ has been previously reported in US TSP samples, though with a greater contribution to ∑_23_OPE than observed here.^31^ *tert-*Butylphenyl diphenyl phosphate was also previously reported,^32^ but the isomer was not specified. Additionally, 4tBPDPP (found in FM 600-treated foam)^42^ was previously reported in the preliminary TSP sample data (*n* = 5 from each site) from these sites used in method validation.^43^ This is the first report of B4tBPPP (found in FM 600-treated foam)^42^ in US TSP samples. This study also represents the first report of “novel” OPEs in PM_2.5_ in the US.

“Traditional” OPEs detected in less than 50% of samples (Table S1) included tri-*o*-tolyl-phosphate (TOTP), tri-*m*-tolyl-phosphate (TMTP), tri-*p*-tolyl-phosphate (TPTP), tris­(2-isopropylphenyl) phosphate (T2IPPP), and tris­(3,5-dimethylphenyl) phosphate (T35DMPP), while “novel” OPEs included 3-isopropylphenyl diphenyl phosphate (3IPPDPP), 4-isopropylphenyl diphenyl phosphate (4IPPDPP), 2-*tert*-butylphenyl diphenyl phosphate (2tBPDPP), 3-*tert*-butylphenyl diphenyl phosphate (3tBPDPP), bis­(2-isopropylphenyl) phenyl phosphate (B2IPPPP), bis­(3-*tert*-butylphenyl) phenyl phosphate (B3tBPPP), and tris­(4-*tert*-butylphenyl) phosphate (T4tBPP). Many of these “novel” OPEs are lesser components of FM 550 or 600,^42^ which explains their low ambient concentrations in samples here.

### Comparison to Previous Studies

What constitutes ∑OPE is inconsistent across studies due to differences in target analyte lists, with the current list containing the largest number of OPEs examined in the US. This can make comparisons of ∑OPE across studies difficult. To make consistent comparisons, target analyte lists were matched as much as possible and the appropriate ∑OPE calculated. Median TSP sample ∑_6_OPE for PC (498 pg m^–3^) and the Port (355 pg m^–3^) was less than median TSP sample ∑_6_OPE reported for Chicago and Cleveland from 2012 to 2014.^30^ It is worth noting that median TSP sample ∑_23_OPE was less than the reported median TSP sample ∑_20_OPE at the Albany, NY airport in 2018,^32^ despite having more OPEs in the target analyte list, which may be due to proximity to sources or regional differences. Additionally, maximum TSP sample ∑_23_OPE ambient concentrations for both sites in this study are less than the maximum TSP sample ∑_8_OPE in Houston, TX in 2013, despite a larger target analyte list. For PM_2.5_ samples, when the target analyte list was matched to those in Houston, maximum ∑_8_OPE in PM_2.5_ from the Port and PC was 2.6 times higher at each site than that reported for PM_2.5_ in Houston.^29^

Tris­[(2*R*)-1-chloro-2-propyl] phosphate (TCPP) was the dominant OPE in this study (Figure 1), detected in 100% of TSP and PM_2.5_ samples at the Port and 100 and 97% of TSP and PM_2.5_ samples at PC, respectively. Median TCPP in TSP samples at PC and the Port (320 and 273 pg m^–3^, respectively; Table 1) was less than those of Chicago (370 pg m^–3^) and Cleveland (387 pg m^–3^) from 2012 to 2014 ^30^ as well as Cleveland (737 pg m^–3^) in 2017 ^31^ and Albany (591 pg m^–3^) in 2018,^32^ but higher than the median in Chicago (199 pg m^–3^) in 2017.^31^ The maximum TCPP ambient concentration in TSP samples at PC (2410 pg m^–3^) was higher than the maximum TCPP ambient concentration reported in Houston (2200 pg m^–3^) in 2013;^29^ however, the maximum TCPP ambient concentration in TSP samples at the Port was lower (1950 pg m^–3^). Conversely, maximum TCPP ambient concentrations in PM_2.5_ samples at PC and the Port (3440 and 3390 pg m^–3^, respectively) were higher than the maximum TCPP ambient concentrations in PM_2.5_ samples reported in Houston in 2013 (870 pg m^–3^).^29^

**Figure 1:**
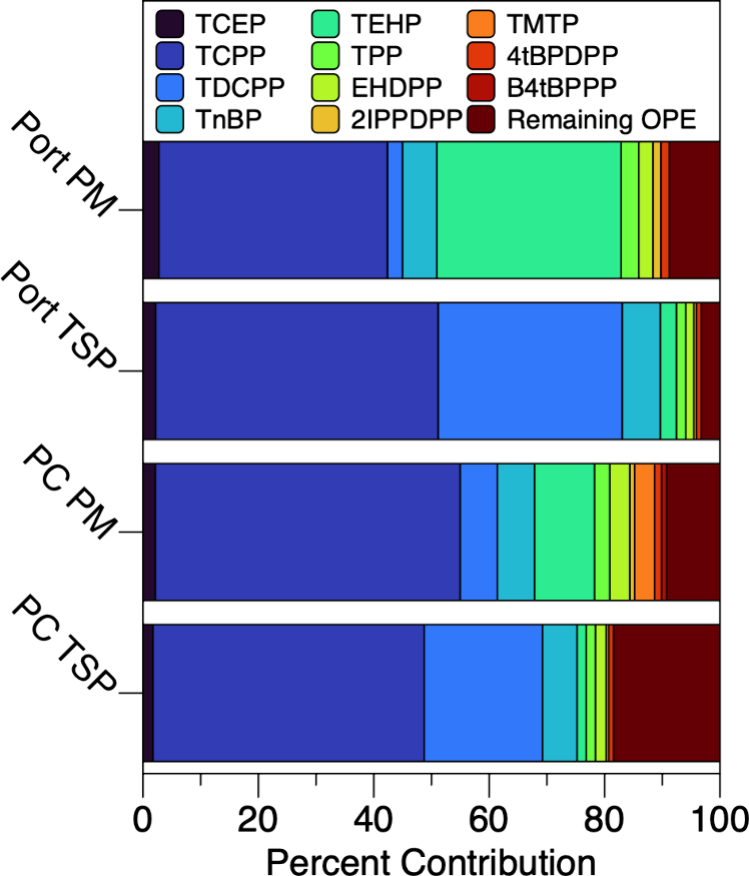
Percent contributions of median ambient concentrations for individual OPEs detected in over 50% of samples and remaining OPEs contributing to ∑Cl-OPE, ∑Alkyl-OPE, ∑Aryl-OPE, and ∑_23_OPE based on the site and size fraction.

Further, median χCl-OPE (the fraction of chlorinated OPEs in a sample, as an analogue for the composition of the sample) were 0.61 and 0.78 in PC TSP and PM_2.5_ samples, respectively, and 0.53 and 0.80 in Port TSP and PM_2.5_ samples, respectively. Chlorinated OPEs (Cl-OPE) frequently dominate observed atmospheric concentrations due to higher usage and environmental persistence^6,20,28–32,44,45^ despite restrictions of tris­(2-chloroethyl) phosphate (TCEP) and tris­(1,3-dichloro-2-propyl) phosphate (TDCPP) in the EU and parts of the US.^46^ For both sites and size fractions, ambient concentrations of TCPP > TDCPP > TCEP. This is consistent with observed prominence of Cl-OPEs in Cleveland in 2012 ^28^ but differed from observations in Chicago,^28,30,31^ Cleveland in 2017,^31^ Albany,^32^ and Houston,^29^ where TCEP was more prominent than TDCPP. This difference may be due to changes in use patterns over time (2023–2024 vs 2018 and older), with increased use of TDCPP as a FR following the ban of penta-BDE^42^ and TCPP replacing TCEP in many applications due to restrictions on TCEP.^47^ Observed differences could also be due to differences in regional OPE profiles. Higher abundance of tri-*n*-butyl phosphate (TnBP), tris­(2-ethylhexyl) phosphate (TEHP), triphenyl phosphate (TPP), and TCEP compared to other OPEs also agreed with previous studies from the Great Lakes Region,^31^ although percent contributions to ∑_23_OPE varied when studies were compared. This further suggests that OPE profiles may vary regionally but may also vary due to source proximity in studies and length of study period/seasonality differences. Seasonal trends in the concentrations of the OPEs and χCl-OPE for both sites and size fractions are explored below. Of these abundant OPEs, the median ambient concentration of TEHP at PC and the Port was higher than reported medians from Albany in 2018 ^32^ and Chicago and Cleveland in 2012,[Bibr ref28] 2012–2014,^30^ and 2017 ^31^ and the average median ambient concentration of TEHP at PC and the Port was higher than reported averages from Houston in 2013.^29^ This increased concentration corresponds to an increase in TEHP use in the market as a flame retardant and plasticizer in recent years.^48^

### Sample Composition

Percent contributions of median ambient concentrations for individual OPEs varied between sites and size fractions (Figure 1). At both sites, larger percent contributions of Cl-OPEs and alkyl-OPEs were observed in PM_2.5_ and TSP samples, respectively. At PC, the percent contribution of aryl-OPE was consistent between size fractions, while it was higher in TSP vs PM_2.5_ samples at the Port. These observations may be in part due to the size fraction preference noted for specific frequently detected OPEs. At both sites, TEHP was almost always below the MDL in PM_2.5_ samples, while it was almost always above the MDL in TSP samples, suggesting a partitioning preference for larger particles, which has been previously observed.^29^ Conversely, TDCPP was detected more frequently above the MDL in PM_2.5_ samples from both sites, suggesting a partitioning preference for smaller particles. These results differ from TDCPP observed in Houston, where ambient concentrations were frequently below the MDL in PM_2.5_ samples^29^ and again could be attributed to use changes (see Comparison to Previous Studies).^42^ OPEs have shown a preference to fine particulate matter (i.e., PM_2.5_),^49–51^ which agrees with higher Σ_23_OPEs ambient concentrations in PM_2.5_ versus TSP samples observed at both sites in this study. Size fraction variability is further explored below.

### Spatial Variability

Σ_23_OPE ambient concentrations in both TSP and PM_2.5_ samples had a positive, statistically significant correlation (Pearson correlation), but concentrations were statistically different (Student's *t* test) between the two sites (Figure 2 and Table S3), despite the two locations (urban and industrial) being less than 6.5 km apart (Figure S1). This combination would suggest that the two sites are influenced by similar sources of OPEs, but a loss process or another dilution between the sites causes significant differences in Σ_23_OPEs. ∑Cl-, ∑Alkyl-, ∑Aryl-, and ∑OPE are frequently used to examine OPEs, especially in previous US-based studies. However, in this study, trends were often driven by individual compounds dominating the overall summation, hiding trends in other individual OPEs or putting summations between the trend of individual OPEs. To this end, individual OPEs were examined.

**Figure 2:**
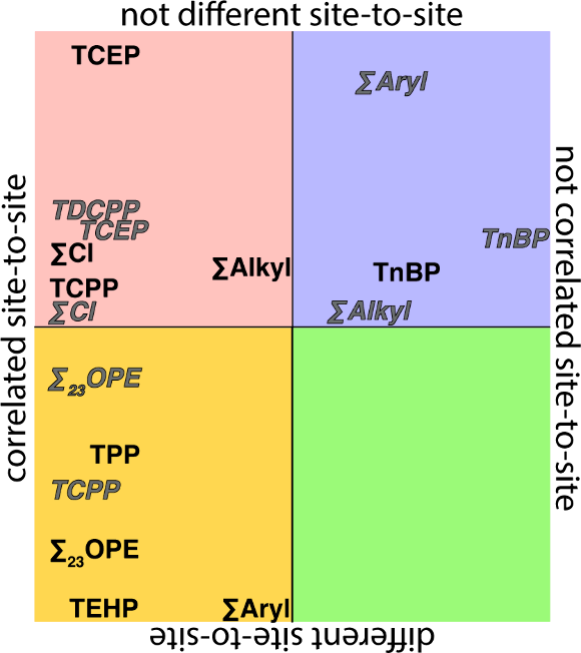
Comparison between sampling sites of Pearson correlation *p*-values and Student's *t*-test *p*-values (Table S3) for individual OPEs and summations for TSP samples (black) and PM_2.5_ samples (gray italicized).

TCEP (TSP and PM_2.5_ samples), TCPP (TSP samples), and TDCPP (PM_2.5_ samples) had a positive, statistically significant correlation and no significant difference in concentrations suggesting the same (or similar) source contributing to both sites without significant loss or dilution over the 6.5 km distance (Figure 2 and Table S3). The consistency in chlorinated OPEs grouped into this category suggests their stability in undergoing atmospheric transport, which has been previously noted.^45^ TCPP (PM_2.5_ samples), TPP (TSP samples), and TEHP (TSP samples) had a positive, statistically significant correlation and a significant difference in concentration suggesting the same source contributing to both sites for each OPE, but with dilution or an otherwise consistent loss process leading to different concentrations between the two sites. Median ambient concentrations of TEHP in Port samples were over three times higher compared to PC, suggesting that TEHP is emitted near the Port and diluted as it travels to PC. This is expected, with several industrial uses in vinyl chlorides and rubber,^52^ causing higher concentrations in more industrial areas (Port) compared to urban areas (PC). Concentrations of TPP were also higher at the Port compared to PC in most date-matched samples, which also agrees with its many industrial uses.^34^ Conversely, TCPP being correlated but statistically different in PM_2.5_ samples (but not in TSP samples) suggests loss in PM_2.5_ that is not seen in larger particles, which bears further study. TnBP (TSP and PM_2.5_ samples ) showed no statistically significant correlation and no significant difference in concentrations, suggesting different sources at each site that contribute similarly to atmospheric concentrations, allowing for the similar observed concentrations. This is not surprising given that TnBP has a variety of uses^53^ and is less likely to undergo long-range atmospheric transport.^35^

In both PM_2.5_ and TSP PC samples, frequently detected OPEs and χCl-OPE were sorted by the day of the week and compared via single-factor ANOVA. There was no statistically significant difference between the days of the week. Additionally, there was no statistically significant difference in Student's *t* tests when weekdays and weekends were compared. This consistency suggests that sources of OPEs to PC are constant and do not “turn off” and “turn on” in a production sense. This agrees with the understanding that OPEs are chemical additives in most consumer products (not bonded to the matrices of products they are added to), allowing for release into different parts of the environment via volatilization, leaching, and/or abrasion.^54–56^ Observed differences between the sites outlined above, despite only being 6.5 km apart, are not unprecedented. A previous study in Albany noted statistical differences in OPEs as little as 2 km apart, but the intensity of signal varied upwind vs downwind of sources.^32^ Variation in upwind vs downwind between these sites (Figure S2) was observed.

At the Port, which is a smaller sample set, only weekdays and weekends were compared. The only statistically significant difference was found between χCl-OPE values in TSP samples. This suggests that while absolute totals of OPEs are not significantly different (possibly due to large variability), the percent composition within the ∑_23_OPE varies on the weekends near an industrial area, which could suggest that different sources of OPEs contribute during the week versus the weekend. A larger sample set from this area (and similar sites) should be used to further investigate.

### OPE Correlations

Individual, frequently detected OPE ambient concentrations within each size fraction (PM_2.5_ or TSP samples) at each site were compared to each other via Pearson correlation to determine if compounds were associated, which can clarify their origins in the atmosphere. If the OPEs share a similar source, a positive, statistically significant correlation will be observed. In PC TSP samples (Table S4), TCEP and TCPP had a positive, statistically significant correlation (*R* > 0.4, *p* < 0.001) with each other, which has been previously observed^29,31^ and agrees with their common use in paint coatings, resins, thermoplastics, textiles, polyurethane foam, and other building materials.^34^ TCEP and TCPP both also had positive, statistically significant correlations with TDCPP, TPP, and TnBP. Following the ban of penta-BDE, TDCPP has seen increased co-use as an FR with FM 550, which contains TPP.^34,42^ This co-usage is further supported here by the observed positive, statistically significant correlation (*R* < 0.4, *p* < 0.01) between TDCPP and TPP. TnBP has many uses, among them as an adhesive and FR in building materials,^34^ potentially explaining the positive, statistically significant correlations (*R* > 0.4, *p* < 0.001) observed with TCEP, TCPP, TDCPP, and TPP. Positive, statistically significant correlations were also observed between EHDPP and TPP, TEHP, and TnBP (*R* > 0.4, *p* < 0.001). These compounds have overlapping usages as FRs and plasticizers in vinyl chlorides and other building materials.^52,57,58^ Additionally, OPEs may be co-used in the same product (one as an FR and one as a plasticizer),^30^ which also accounts for observed correlations. Correlations observed in previous studies are noted in Table S4.

In TSP samples from the Port (Table S5), TCEP, TCPP, TEHP, TnBP, and TPP were compared due to detection frequency above the MDL. Between these five OPEs, the same positive, statistically significant correlations were observed as in PC TSP samples (*R* > 0.4, *p* < 0.001), except for TnBP and TEHP, which had a negative, statistically significant correlation. This suggests similar, though not the same, sources of correlated OPEs between sites. In PC and Port PM_2.5_ samples (Tables S6 and S7), TCEP, TCPP, TDCPP, and TnBP were compared due to detection frequency above the MDL. Between these four compounds, all associations had *R* > 0.4 and *p* < 0.05, except TDCPP vs TnBP in Port samples, which was not statistically significant. Correlations observed in previous studies are noted in Tables S5–S7.

### Size Fraction Correlations

TCEP (PC) and TCPP (PC and Port) had a positive, statistically significant correlation (Pearson correlation) and no significant difference in concentrations (Student's *t* test) (Figure 3 and Table S8) when size fractions were compared, suggesting that the sources of these OPEs partition indiscriminately as there is no dilution of concentrations with the addition of PM larger than 2.5 μm. TCPP being correlated and statistically different in PM_2.5_ (but not in TSP) samples at each site combined with being correlated but not statistically different when comparing TSP and PM_2.5_ ambient concentrations (and TCPP almost always being higher in Port samples) suggests that there is a second source of TCPP in PM_2.5_ at the Port that does not affect PC. If this source is associated with industry near the Port of Providence, this manufacturing may be releasing finer particles, which influence the concentrations at the Port but not at PC.

**Figure 3:**
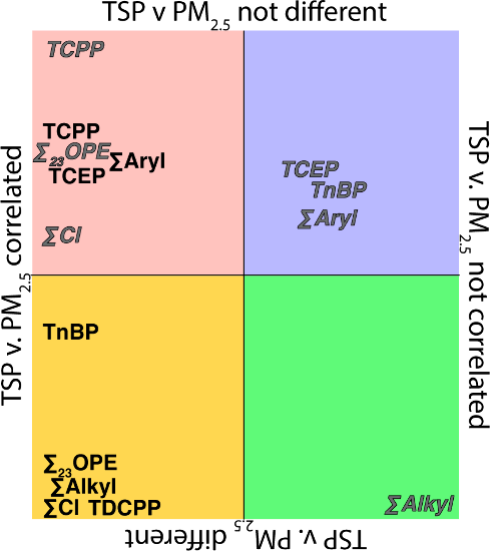
Comparison between sample size fractions (PM_2.5_ vs TSP) of Pearson correlation *p*-values and Student's *t*-test *p*-values (Table S8) for individual OPEs and summations for PC samples (black) and Port samples (gray italic).

TnBP (PC) and TDCPP (PC) had a positive, statistically significant correlation and significant difference in concentration, suggesting that sources of these OPEs show preference for PM_2.5_, with higher concentrations in PM_2.5_ compared with TSP samples. This further supports previous observations made in this study regarding TDCPP (Sample Composition *)*. TnBP (Port) and TCEP (Port) had no statistically significant correlation and no significant difference in concentrations suggesting similar but unrelated sources of fine and coarse particles containing these compounds. Differences in TnBP further support previous observations made in this study (Spatial Variability). Previously, no correlation was found between PM_2.5_ and TSP samples for TCPP, but was observed for TnBP.^29^

### Association with Meteorological Parameters

Particle-bound OPEs should be susceptible to wet and dry deposition,^59^ but recent studies have found particle-bound OPEs (especially PM_2.5_) increasing in the rainy season.^34,44^ In this study, PC samples had no statistically significant difference when rain and nonrain samples were compared via Student's *t* test (Table S9), except for TEHP and χCl-OPE in TSP samples and TCPP in PM_2.5_ samples, the latter two with higher mean atmospheric concentrations in rain samples. Rain is more effective at scavenging coarse particles (PM greater than 2.5 μm) versus fine particles (PM_2.5_),^60^ which may explain decreasing TEHP. Increasing concentrations in rain samples for TCPP in PM_2.5_ samples bear further investigation.

While meteorological factors other than temperature have previously shown to have a negligible effect on concentrations of some semivolatile organic compounds (SVOCs) in the air,^61^ factors such as barometric pressure (BP), solar radiation (SR), relative humidity (RH), and wind speed (WS) have been shown to have an effect on OPE atmospheric concentrations.^33,35,45,59^ Frequently detected individual OPE compounds in both PM_2.5_ and TSP PC samples were compared to meteorological parameters to see if there was an association via Pearson correlation (Table S10). There was no statistically significant correlation found for BP and the investigated OPEs, despite BP enhancing particle-bound OPEs previously.^35^ SR, which is involved in OPEs reacting with hydroxyl radicals in lab-based studies,^45^ only had a positive, statistically significant correlation with TDCPP in PM_2.5_ samples. Additionally, there was no statistically significant correlation for RH in TSP samples except for TnBP (*R* < 0.4, *p* < 0.05), but there was a positive, statistically significant correlation found in PM_2.5_ samples between RH and TCEP, TCPP, and TDCPP as well as ∑Cl and ∑_23_OPE (*R* > 0.4, *p* < 0.05) and ∑Alkyl (*R* < 0.4, *p* < 0.05). Correlations between RH and TCEP and TCPP in PM_2.5_ samples have been previously observed in urban South China, where RH was found to strongly influence OPEs in PM_2.5_, allowing for greater sorption.^33^ This could also explain the higher concentrations of TDCPP and ∑_23_OPE observed in PM_2.5_ samples compared to TSP samples in this study.

A negative, statistically significant correlation was found for WS in both TSP and PM_2.5_ samples for TCPP, ∑Aryl, ∑_23_OPE (*R* < 0.4, *p* < 0.05), and ∑Cl (*R* < 0.4, *p* < 0.05). An additional negative, statistically significant correlation with WS was noted for TnBP, TPP, EHDPP, and ∑Alkyl in TSP (*R* > 0.4, *p* < 0.05), TEHP in TSP (*R* < 0.4, *p* < 0.05), and TDCPP and ∑Alkyl in PM_2.5_ (*R* < 0.4, *p* < 0.05). This may indicate short-range atmospheric transport influencing OPE ambient concentrations, which may further explain trends seen in this report for TCPP, TnBP, TPP, and TEHP.

A positive, statistically significant correlation was found in both PM_2.5_ and TSP samples for temperature and TnBP (*R* < 0.4, *p* < 0.005) as well as TCPP, χCl-OPE, ∑Cl, and ∑_23_OPE (*R* > 0.4, *p* < 0.01). An additional positive, statistically significant correlation was found for temperature and TPP (*R* < 0.4, *p* < 0.01) and TCEP (*R* > 0.4, *p* < 0.05) in TSP samples and ∑Alkyl (*R* < 0.4, *p* < 0.01) in PM_2.5_ samples. Correlations between TnBP and TCPP with temperature were previously reported in PM_2.5_ samples in South China, suggesting that these OPEs are evaporating from contaminated surfaces and binding to particles in the air,^33,35,59^ and agree with previously reported behaviors for other SVOCs.^61^

### Seasonal Trends

Due to observed strong correlations with temperature for several frequently detected OPEs as well as summations (Associations with Meteorological Parameters; Table S10), seasonal trends for PM_2.5_ and TSP samples were explored based on meteorological seasons (autumn: Sept. through Nov.; winter: Dec. through Feb.; spring: Mar. through May; summer: June through Aug.). While there are limitations to examining this variability with 1 year of samples, previous studies also compared these trends with 1 year of data.^31,33^ At PC (Figure 4), there was a statistically significant difference (Student's *t* test; *p* < 0.01) for ∑_23_OPE in PM_2.5_ samples when comparing the seasons to each other, except autumn vs summer and winter vs spring (Tables S11 and S12). A similar trend was observed in TSP samples, except that there was no statistically significant difference between winter vs summer TSP samples. χCl-OPE was also compared seasonally to compare sample composition, with a statistically significant difference found between summer vs each of the other three seasons in both PM_2.5_ and TSP samples, with an additional statistically significant difference between winter vs spring in TSP samples.

**Figure 4:**
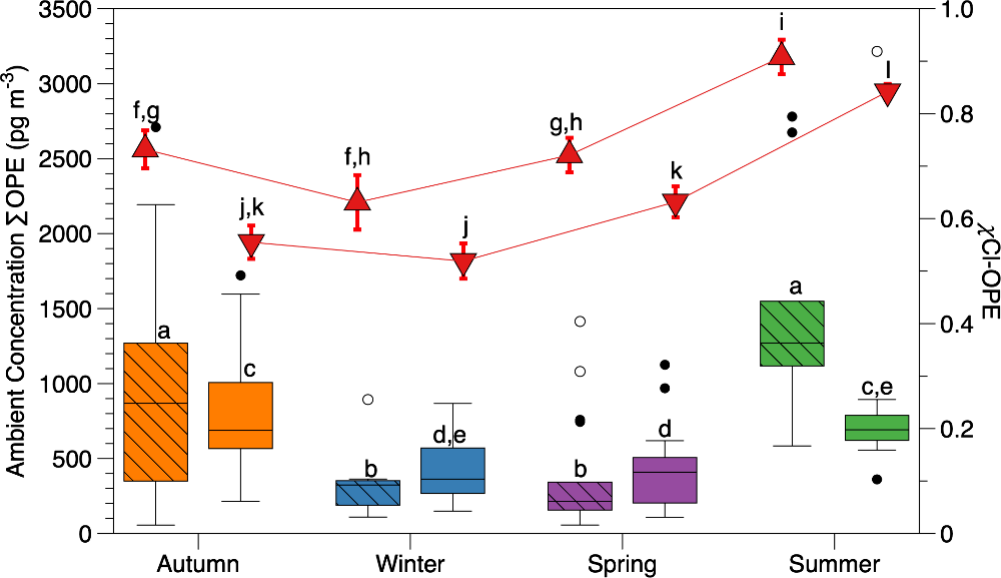
Box and whisker plots for ∑_23_OPE in PM_2.5_ (striped) and TSP (solid) samples from PC based on the meteorological season as well as χCl for PM_2.5_ (red upward triangle) and TSP (red downward triangle), with letters showing statistical differences between data sets.

For ∑Cl-OPE, only winter vs spring was not statistically significantly different in both TSP and PM_2.5_ samples (Table S12 and Figure S3). This is consistent with the predominance and seasonal trends observed for TCPP (and TDCPP in PM_2.5_; Table S11 and Figure S3). TCEP was the most consistent between seasons (Table S11 and Figure S3) but contributed the least to ∑Cl-OPE, therefore providing the least influence. Seasonal trends diverged greatly in ∑Alkyl-OPE, with a statistically significant difference between autumn vs winter, autumn vs spring, and spring vs summer in PM_2.5_ samples, but only in autumn vs spring and autumn vs summer in TSP samples (Table S12 and Figure S3). For PM_2.5_, this is consistent with observed TnBP trends, which had the greatest contribution to ∑Alkyl-OPE. For TSP samples, the overall trend does not lean toward either TnBP or TEHP, which both have different seasonal trends (Table S11 and Figure S3), which agrees with previous observations in this report of differing sources. Finally, in ∑Aryl-OPE, only autumn vs spring showed a statistically significant difference in both TSP and PM_2.5_ samples, while there was also a statistically significant difference between spring vs summer in TSP samples (Table S12 and Figure S3). This followed the same trend as TPP (Table S11 and Figure S3), despite it not being a very large contribution to ∑Aryl-OPE in TSP samples. Trends in ∑Aryl-OPE in PM_2.5_ were a combination of compounds not frequently detected above the MDL. At the Port, only spring vs summer could be compared for PM_2.5_ and agreed with statistically significant differences observed in PC samples.

Previous studies have observed a statistically significant difference between winter vs summer in TSP samples for ∑Cl-, ∑Aryl-, ∑Alkyl-, and ∑OPE.^31^ This difference was observed only for ∑Cl-OPE in this study, further suggesting regional differences in ambient concentrations. No US-based study has previously reported PM_2.5_ seasonal trends, but in China, concentrations were highest in late spring/early summer and lowest in fall,^33^ also suggesting regional differences in OPE ambient concentrations. The authors acknowledge that there is a limitation in looking at seasonal trends with 1 year of data vs comparing seasons across multiple years, and efforts are ongoing to see if these trends hold year-to-year.

In addition to 1-in-6-day sampling, week-long sampling intensives were carried out in the fall, spring, and summer at PC as well as at the Port in the spring and then compared to the median 1-in-6-day OPE ambient concentrations for that season (Figure 5). In both fall and spring intensives (Figure 5a,b), while the median ∑_23_OPE for the intensive period and the median for 1-in-6 samples collected over the season were similar, there was large variability in the individual OPEs day-to-day, as previously reported,^29^ and within these medians. Depending on sampling strategies, this high variability in individual concentrations (as well as “high” ∑_23_OPE days like those observed in fall) could lead to chronic under- or overreporting of ambient concentrations of OPEs, which could lead to under- or overestimated levels of exposure to OPEs. While the authors admit that daily, long-term sampling is not sustainable due to time and cost, the inclusion of more intensive sampling strategies in the monitoring of OPEs should be considered to better understand other regional compositions.

**Figure 5:**
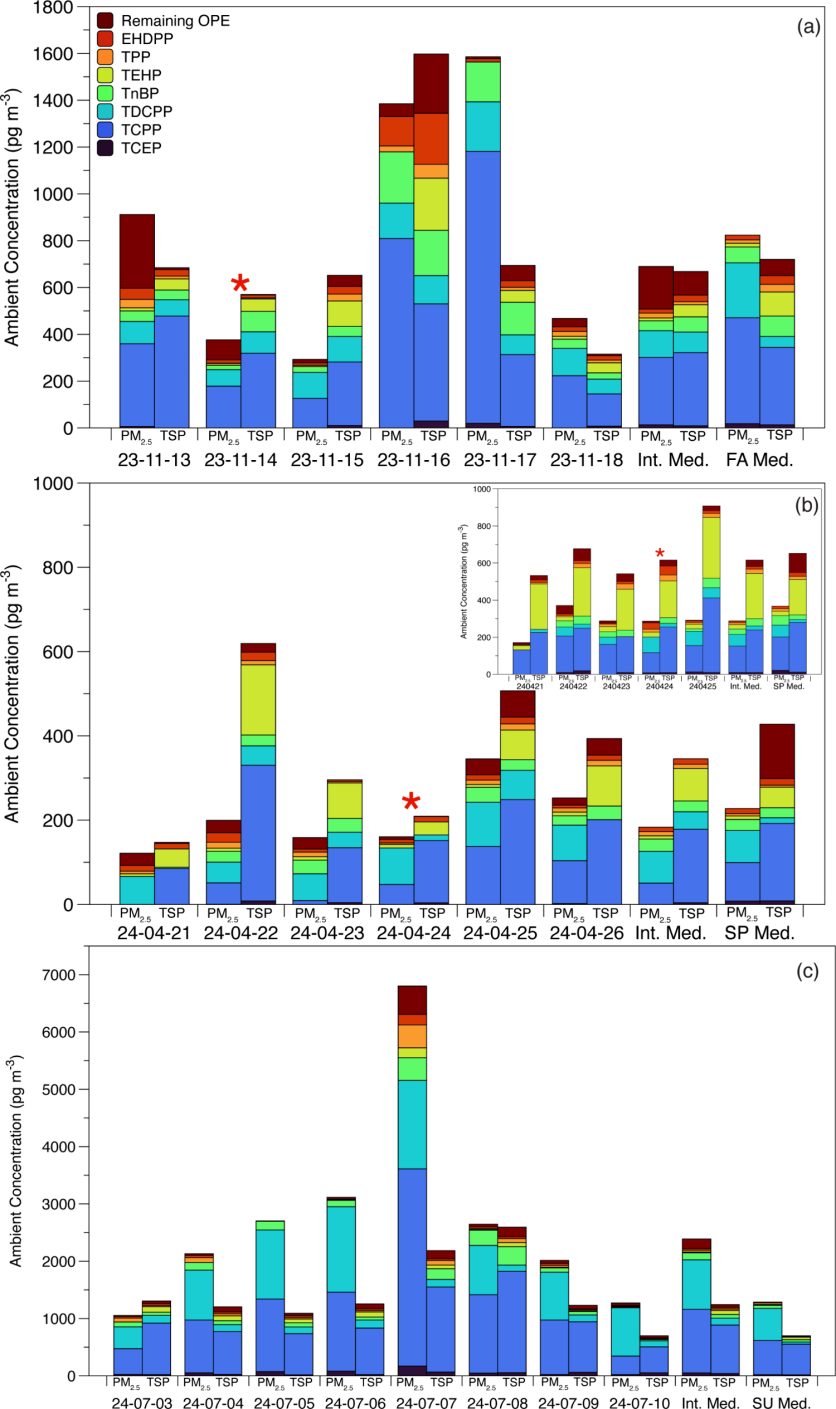
Intensive day-to-day samples vs median for the intensive (Int. Med.) and median for the season for individual frequently detected OPEs in PM_2.5_ samples (left bars) and TSP samples (right bars) for (a) autumn (FA) at PC, (b) spring (SP) at PC (Port inset), and (c) summer (SU) night samples at PC. The red star indicates the “regularly scheduled” 1-in-6-day sample.

In the summer intensive, night samples (10 h) were taken in lieu of 24 h samples (Figure 5c). Median ∑_23_OPE ambient concentrations were nearly twice as high for the intensive period compared to the 1-in-6-day 24 h samples. Higher ambient concentrations of certain OPEs in limited night samples have been previously reported in Houston TSP samples.^29^ To the best of the authors’ knowledge, this represents the first report of OPE PM_2.5_ ambient concentrations in night samples. Additionally, high night-to-night variability was also observed, with maximum ∑_23_OPE (all sites and size fractions) in this study being observed on the night of July 7 in PM_2.5_ at PC. Additionally, the third and second highest ∑_23_OPE in PC TSP samples were observed from July 7 and 8, respectively. NOAA HYSPLIT^62,63^ back trajectories show that the air mass on the night of July 7 moved differently compared to the other nights in this intensive (Figure S4), passing through more and different urban areas, which may account for the higher observed concentrations. At the Port, where a more limited number of night samples were taken (*n* = 3) for both size fractions, the second and third highest ∑_23_OPE for TSP samples were observed on July 4 and 9 and the third highest ∑_23_OPE for PM_2.5_ samples was observed on July 9. These high concentrations may be due to a significant amount of OPEs released overnight compared to a more diluted 24 h period, despite lower temperatures leading to lower volatility, but may also be due to the atmospheric boundary layer trapping the OPEs overnight, limiting transport from sources when volatilized during the warmer daytime hours. Either scenario bears future study.

*Data note:* Preliminary TSP sample ambient concentrations from Feb. 12 through March 7, 2024 (*n* = 5 at each site) were previously reported as part of method validation^43^ but are included here as part of the larger, year-long trend analysis and examination of relationships with meteorological parameters and among size fractions.

## Conclusions

Twenty-three OPEs have been reported in 89 TSP samples and 89 PM_2.5_ samples taken over the course of a year at two sites in Providence, RI, marking the first reports of OPEs in the US in 5 years. Detected in more than 50% of samples, B4tBPPP was reported in both size fractions for the first time in the US while 2IPPDPP, 4IPPDPP, and 4tBPDPP were reported in PM_2.5_ samples for the first time in the US. Additional, less frequently detected novel OPEs were also reported in both size fractions for the first time in the US, including 3IPPDPP, 2tBPDPP, 3tBPDPP, B2IPPPP, and B3tBPPP, with T4tBPP also reported in PM_2.5_ samples for the first time in the US. The dominant OPE in this study was TCPP, which agrees with previous studies, though median ambient concentrations in TSP samples are not as high as those previously reported. Conversely, TEHP median ambient concentrations in TSP samples exceeded previous reports. Correlations between individual OPEs suggest similar sources contributing various OPEs to the atmosphere while highlighting the complex nature of the use of OPEs in consumer products and regional differences in the characteristics of OPE profiles. The reported PM_2.5_ sample correlations are among the first to be reported in the US. Meteorological associations varied, with temperature correlations agreeing with previous observations of OPE volatility and entrance to the atmosphere and RH agreeing with previous enhancement in PM_2.5_ partitioning. Additionally, there was no indication of precipitation scavenging of the OPEs from the atmosphere when rain and nonrain samples were compared, with the exception of TEHP in TSP samples, which had a lower average ambient concentration in rain samples compared to nonrain samples. Conversely, TCPP ambient concentrations in PM_2.5_ samples were significantly higher in rain samples compared to nonrain samples, which bears further investigation.

Differences in the OPE profiles compared to other studies as well as differences in seasonal observations suggest that there are regional differences in OPE profiles or changes over time in the prominence of OPEs (the last US-based study was in 2018), or a combination thereof. High concentrations of OPEs in night samples bear further study here and in other regions. The two sites included in this study had some significant differences, despite being less than 6.5 km apart. Large differences across a small spatial distance suggest that traditional sampling strategies may not adequately capture OPE profiles, especially in complex, mixed-use urban areas. Additionally, high day-to-day variability during intensive sampling periods suggests that more intensive sampling efforts should be undertaken to ensure that OPE exposures are not being chronically over- or underreported via traditional 1-in-6 or 1-in-12-day sampling schemes. Sampling in this region and analysis of these compounds should continue as a better understanding of exposures to flame retardants and plasticizers, especially in urban areas, is important to human health, as exposures to plastics and other products containing these compounds will continue for decades to come.

## Methods

### Sampling

PM_2.5_ and TSP samples were collected at PC (urban; 41.842716, −71.438802) and the Port of Providence (industrial; ∼6.4 km SE of PC; 41.795139, −71.397906) as part of the Friar Air Monitoring Network (FriAir Net; Figure S1) using Tisch active high volume PM_2.5_ and TSP samplers (TE-PM2.5PLUS and TE-PNY1123, respectively; Tisch Environmental, Cleves, OH, USA) at each site. The PC site also has a MetOne Ready Weather Station (MetOne Instruments, Grants Pass, OR, USA), which takes measurements every 15 min of temperature, wind direction, WS, BP, RH, SR, and precipitation. Samples from PC were collected between September 2023 and September 2024 for 24 h (approximately 0600 to 0600) on a 1-in-6-day sampling schedule, except for once a season week-long intensives where daily sampling took place (PC TSP *n* = 66; PC PM_2.5_ *n* = 63), while Port samples were taken on the same schedule as PC samples between February and September 2024 (Port TSP *n* = 23; Port PM_2.5_ *n* = 26). During the summer intensive, daily nighttime samples (2000 to 0600) were taken. All samplers were calibrated prior to deployment, and samplers used ambient temperature and BP to calculate the ambient volume of air sampled. Field blanks were taken approximately every 4 weeks. Samples were collected on quartz fiber filters (QFF) prepared as previously described.^43^

### Chemicals, Materials, and Chemical Analysis

All solvents, OPE standards, and other materials were purchased from commercial vendors as previously described.^43^ Aliquots of known area of sampled QFF were taken based on size fraction. QFF aliquots were prepared and extracted as previously described.^43^ Samples were extracted in batches of five to six, including an associated field blank (date matched to samples) or laboratory blank, which was used for blank correction as needed.

Sample extracts were analyzed for a total of 31 OPEs via gas chromatography–mass spectrometry (GC-MS). A full list of names and abbreviations is given in the Supporting Information (Table S13). The GC-MS method, analyte identification, and quantitation methods have been previously described and validated.^43^ For tri-*n*-butyl phosphate (TnBP), *m*/*z* 211 was used for quantitation in most samples, instead of *m*/*z* 99, due to sample interferences with the same ion at approximate retention time. A check standard (near the midpoint of the calibration curve) was run on the GC-MS before and after each batch to ensure calibration validation, and a solvent blank was run before and after each check standard. In the event that chemical concentrations from GC-MS were higher than the highest point of the calibration curve, samples were rerun on a calibration curve with higher concentrations. Method detection limits (MDLs) were previously reported^43^ and are summarized in Table S12.

### Statistical Analyses

Pearson correlation coefficients and *p*-values were determined using the regression function in Analysis ToolPak in Microsoft Excel. Student's *t* tests and single-factor ANOVA were also carried out using Analysis ToolPak in Microsoft Excel. Where appropriate, samples were date-matched for comparison, and dates where data were missing from one category were removed from analysis. If OPE concentrations were below the MDL, then chemical concentrations were tentatively assigned and used in calculations and interpretations as appropriate based on a previous report.^29^

## Supplementary Material



## Notes

(Table S1) Median, average, minimum and maximum, and detection frequency of all OPEs examined; (Table S2) average surrogate recoveries; (Tables S3–S8 and S10) Pearson correlation coefficients and *p*-values for correlation; (Tables S9, S11, and S12) *p*-values for two-tailed Student's *t* tests; (Table S13) list of compound names, abbreviations, method detection limits, and chemical structures for OPEs; (Figure S1) map of sampling locations; (Figure S2) results of NOAA HYSPLIT cluster analysis; (Figure S3) box and whisker plots; (Figure S4) NOAA HYSPLIT back trajectories (PDF)
