# Correction to “Spatioseasonal Variability and Correlations of Particle-Bound Organophosphate Esters in Providence, Rhode Island”

**DOI:** 10.1021/acsomega.6c05635

**Published:** 2026-07-10

**Authors:** Annie Gathof, Leart Jahaj, Savannah Patalano, Adelaide E. Clark

The authors wish to acknowledge two errors that were discovered after the publication of this report.

1. An error was noticed in the labeling of Figure 1 in the original manuscript. The TSP samples for each site had been labeled at PM_2.5_ and vice versa. All interpretations of the figure in the manuscript were correct. The corrected Figure 1 is below.

**Figure 1:**
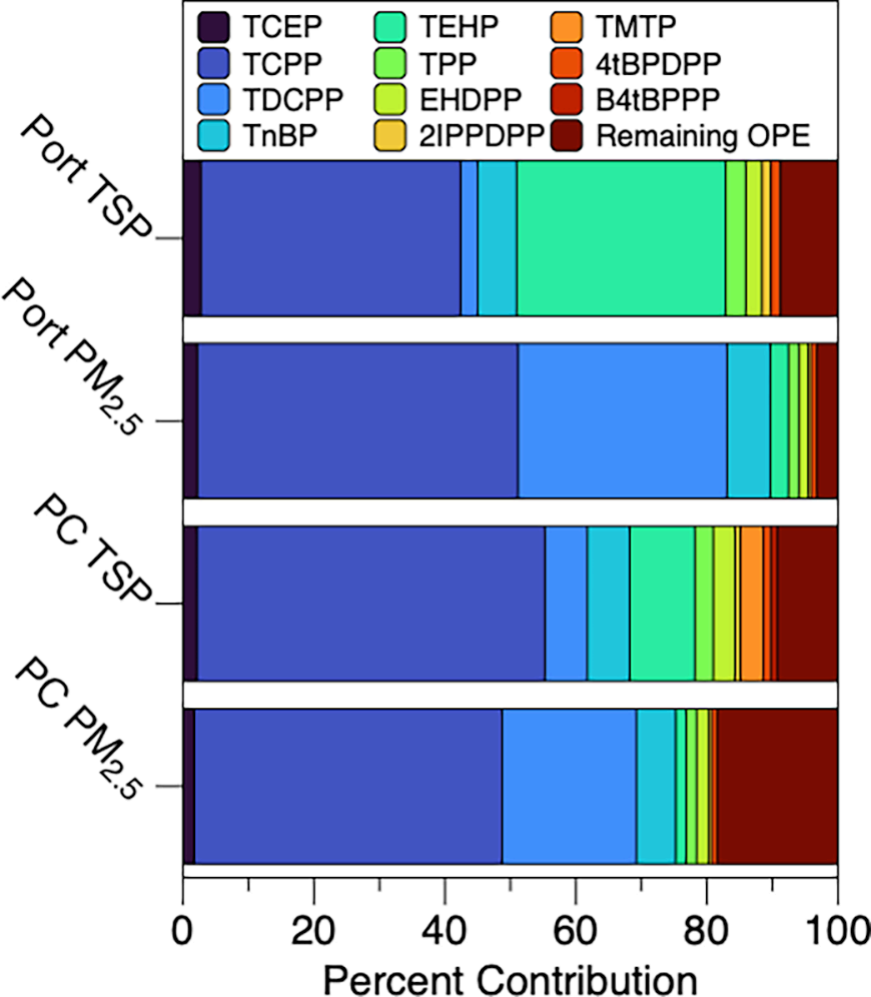
Percent contributions of median ambient concentrations for individual OPEs detected in over 50% of samples and remaining OPEs contributing to ∑Cl-OPE, ∑Alkyl-OPE, ∑Aryl-OPE, and ∑_23_OPE based on site and size fraction.

This also results in a change to the TOC and Abstract graphic (which contains Figure 1):
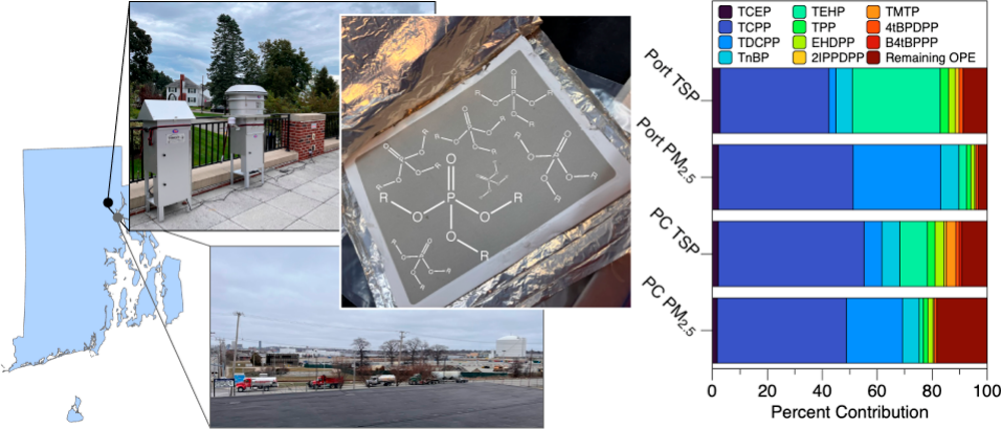


2. There were small mistakes in the sample volumes for a handful of PC samples, but one TSP sample had used the volume for a night sample (10 h) instead of a 24 h sample. This changed the final ambient concentrations of this one sample, which in turn affected calculated means, medians, and some statistical analyses.

None of these changes affect the overall conclusions made in the original report, but the following statements are corrected:

a. (*Page 61598, Original Statement*): The maximum TCPP ambient concentration in TSP samples at PC (2410 pg m^–3^) was higher than the maximum TCPP ambient concentration reported in Houston (2200 pg m^–3^) in 2013;^29^ however, the maximum TCPP ambient concentration in TSP samples at the Port was lower (1950 pg m^–3^).
  (*Corrected Statement*): The maximum TCPP ambient concentration in TSP samples at PC was 1770 pg m^–3^, making it lower than the maximum TCPP ambient concentration reported in Houston (2200 pg m^–3^) in 2013^29^ but similar to the Port value (1950 pg m^–3^).
b. (*Page 61598, Original Statement*): Further, median χCl-OPE (the fraction of chlorinated OPEs in a sample, as an analogue for the composition of the sample) were 0.61 and 0.78 in PC TSP and PM_2.5_ samples, respectively, and 0.53 and 0.80 in Port TSP and PM_2.5_ samples, respectively.
  (*Corrected Statement*): Further, median χCl-OPE values (the fraction of chlorinated OPEs in a sample, as an analogue for OPE sample composition) were 0.64 and 0.79 in PC TSP and PM_2.5_ samples, respectively, and 0.53 and 0.80 in Port TSP and PM_2.5_ samples, respectively.
c. (*Page 61599, Original Statement*): TCEP (TSP and PM_2.5_ samples), TCPP (TSP samples), and TDCPP (PM_2.5_ samples) had a positive, statistically significant correlation and no significant difference in concentrations suggesting the same (or similar) source contributing to both sites without significant loss or dilution over the 6.5 km distance (Figure 2 and Table S3).
  (*Corrected Statement*): TCEP (TSP samples) and TDCPP (PM_2.5_ samples) had a positive, statistically significant correlation and no significant difference in concentrations, suggesting the same (or similar) source contributing to both sites without significant loss or dilution over the 6.5 km distance (Figure 2 and Table S3).
d. (*Page 61599, Original Statement*): TCPP (PM_2.5_ samples), TPP (TSP samples), and TEHP (TSP samples) had a positive, statistically significant correlation and a significant difference in concentration suggesting the same source contributing to both sites for each OPE, but with dilution or an otherwise consistent loss process leading to different concentrations between the two sites.
  (*Corrected Statement*): TCPP (PM_2.5_ and TSP samples), TPP (TSP samples), and TEHP (TSP samples) had a positive, statistically significant correlation and significant difference in concentration, suggesting the same source contributing to both sites for each OPE, but with dilution or otherwise consistent loss process leading to different concentrations between the two sites.
e. (*Page 61599, Original Statement*): Conversely, TCPP being correlated, but statistically different in PM_2.5_ samples (but not in TSP samples) suggests loss in PM_2.5_ that is not seen in larger particles, which bears further study.
  (*Corrected Statement*): [Statement deleted.]
f. (*Page 61599, Original Statement*): Following the ban of penta-BDE, TDCPP has seen increased co-use as a FR with FM 550, which contains TPP.^34, 42^ This co-usage is further supported here by the observed positive, statistically significant correlation (*R* < 0.4, *p* < 0.01) between TDCPP and TPP.
  (*Corrected Statement*): [Statement deleted.]
g. (*Page 61600, Original Statement*): In this study, PC samples had no statistically significant difference when rain and nonrain samples were compared via Student’s *t* test (Table S9), except for TEHP and χCl-OPE in TSP samples and TCPP in PM_2.5_ samples, the latter two with higher mean atmospheric concentrations in rain samples.
  (*Corrected Statement*): In this study, PC samples had no statistically significant difference when rain and nonrain samples were compared via Student’s *t* test (Table S9), except for TEHP and χCl-OPE in TSP samples, the latter with higher mean atmospheric concentrations in rain samples.
h. (*Page 61600, Original Statement*): Increasing concentrations in rain samples for TCPP in PM_2.5_ samples bears further investigation.
  (*Corrected Statement*): [Statement deleted.]
i. (*Page 61600, Original Statement*): SR, which is involved in OPEs reacting with hydroxyl radicals in lab-based studies,^45^ only had a positive, statistically significant correlation with TDCPP in PM_2.5_ samples.
  (*Corrected Statement*): SR, which is involved in OPEs reacting with hydroxyl radicals in lab-based studies,^45^ only had a positive, statistically significant correlation with TDCPP in PM_2.5_ samples and χCl-OPE in TSP samples.
j. (*Page 61600, Original Statement*): Additionally, there was no statistically significant correlation for RH in TSP samples except for TnBP (*R* < 0.4, *p* < 0.05), but there was a positive, statistically significant correlation found in PM_2.5_ samples between RH and TCEP, TCPP, and TDCPP as well as ∑Cl and ∑_23_OPE (*R* > 0.4, *p* < 0.05) and ∑Alkyl (*R* < 0.4, *p* < 0.05).
  (*Corrected Statement*): Additionally, there was no statistically significant correlation for RH in TSP samples except for TnBP, TCPP, ∑Cl and ∑_23_OPE (*R* < 0.4, *p* < 0.05), but there was a positive, statistically significant correlation found in PM_2.5_ samples between RH and TCPP and between RH and TDCPP as well as between ∑Cl and ∑_23_OPE (*R* > 0.4, *p* < 0.05) and between ∑Cl and ∑Alkyl (*R* < 0.4, *p* < 0.05).
k. (*Page 61600, Original Statement*): A negative, statistically significant correlation was found for WS in both TSP and PM_2.5_ samples for TCPP, ∑Aryl, ∑_23_OPE (*R* < 0.4, *p* < 0.05), and ∑Cl (*R* < 0.4, *p* < 0.05). An additional negative, statistically significant correlation with WS was noted for TnBP, TPP, EHDPP, and ∑Alkyl in TSP (*R* > 0.4, *p* < 0.05), TEHP in TSP (*R* < 0.4, *p* < 0.05), and TDCPP and ∑Alkyl in PM_2.5_ (*R* < 0.4, *p* < 0.05).
  (*Corrected Statement*): A negative, statistically significant correlation was found for WS in both TSP and PM_2.5_ samples for ∑Aryl and ∑_23_OPE (*R* > 0.4, *p* < 0.05), and TCPP and ∑Cl (*R* < 0.4, *p* < 0.05 for PM_2.5_, *R* > 0.4, *p* < 0.001 for TSP). Additional negative, statistically significant correlation with WS was noted for TnBP, TPP, and ∑Alkyl in TSP (*R* > 0.4, *p* < 0.05), TCEP, EHDPP, and TEHP in TSP (*R* < 0.4, *p* < 0.05), and TDCPP and ∑Alkyl in PM_2.5_ (*R* < 0.4, *p* < 0.05).
l. (*Page 61600–61601, Original Statement*): A positive, statistically significant correlation was found in both PM_2.5_ and TSP samples for temperature and TnBP (*R* < 0.4, *p* < 0.005) as well as TCPP, χCl-OPE, ∑Cl, and ∑_23_OPE (*R* > 0.4, *p* < 0.01). An additional positive, statistically significant correlation was found for temperature and TPP (*R* < 0.4, *p* < 0.01) and TCEP (*R* > 0.4, *p* < 0.05) in TSP samples and ∑Alkyl (*R* < 0.4, *p* < 0.01) in PM_2.5_ samples.
  (*Corrected Statement*): A positive, statistically significant correlation was found in both PM_2.5_ and TSP samples for temperature and TCPP, χCl-OPE, ∑Cl, and ∑_23_OPE (*R* > 0.4, *p* < 0.01). An additional positive, statistically significant correlation was found for temperature and TCEP (*R* > 0.4, *p* < 0.001) in TSP samples and TnBP and ∑Alkyl (R < 0.4, *p* < 0.05) in PM_2.5_ samples.
  (*Added Statement*): A negative, statistically significant correlation was found in TSP samples between temperature and TEHP, meaning concentrations decrease with increasing temperature, which bears further investigation.
m. (*Page 61601, Original Statement*): At PC (Figure 4), there was a statistically significant difference (Student’s *t* test; *p* < 0.01) for ∑_23_OPE in PM_2.5_ samples when comparing the seasons to each other, except autumn vs summer and winter vs spring (Tables S11 and S12).
  (*Corrected Statement*): At PC (Figure 4), there was a statistically significant difference (Student’s *t* test; *p* < 0.01) for ∑_23_OPE in PM_2.5_ and TSP samples when comparing the seasons to each other, except autumn vs summer and winter vs spring (Tables S11 and S12).
n. (*Page 61601, Original Statement*): A similar trend was observed in TSP samples, except that there was no statistically significant difference between winter vs summer TSP samples.
  (*Corrected Statement*): [Statement deleted.]
o. (*Page 61601, Original Statement*): Finally, in ∑Aryl-OPE only autumn vs spring showed a statistically significant difference in both TSP and PM_2.5_ samples, while there was also a statistically significant difference between spring vs summer in TSP samples (Table S12 and Figure S3).
  (*Corrected Statement*): Finally, in ∑Aryl-OPE only autumn vs spring showed a statistically significant difference in both TSP and PM_2.5_ samples, while there was also a statistically significant difference between autumn vs summer in TSP samples (Table S12 and Figure S3).

The corrected statistical values are summarized in the corrected Supporting Information, included here.

Table 1 is updated based on new calculated median and maximum ambient concentrations.

**Table 1:** Median (Med) and Maximum (Max) Ambient Concentrations (pg m^–3^) and Detection Frequencies (DF, %) of OPEs Detected in More than 50% of Samples, by Size Fraction and Sampling Site^a^

	*Providence College*						*Port of Providence*					
	PM_2.5_ (*n* = 63)			TSP (*n* = 66)			PM_2.5_ (*n* = 26)			TSP (*n* = 23)		
	Med	Max	DF	Med	Max	DF	Med	Max	DF	Med	Max	DF
TCEP	13	170	70	13	65	82	19	64	69	19	49	91
TCPP	353	3440	97	320	1770	100	422	3390	100	273	1450	100
TDCPP	154	1540	100	39	142	86	275	2400	92	<MDL	55	78
**∑Cl-OPE**	528	5160	100	388	1930	100	698	5860	100	285	1480	100
TnBP	45	462	90	39	322	95	57	434	88	41	211	91
TEHP	<MDL	174	79	60	242	98	<MDL	56	96	220	327	100
**∑Alkyl-OPE**	53	568	98	124	416	100	75	455	100	254	379	100
TPP	<MDL	400	95	17	139	92	<MDL	114	77	21	95	91
EHDPP	<MDL	185	97	20	218	97	<MDL	84	92	<MDL	48	91
2IPPDPP	<MDL	165	59	<MDL	27	59	<MDL	28	59	<MDL		96
TMTP				<MDL	91	64						
4tBPDPP	<MDL	53	83	<MDL	37	85	<MDL	24	65	<MDL	32	78
B4tBPPP				<MDL	18	53						
**∑Aryl-OPE**	54	1080	100	64	530	100	51	276	100	78	163	100
**∑**_**23**_ **OPE**	758	6800	100	600	2590	100	889	6270	100	689	1950	100

a <MDL indicates that more than half of the detected ambient concentrations were less than the method detection limits.

Figures 2 and 4 were also updated. For Figure 2, most compounds and summations are still within the same quadrant as in the original publication but may have moved position slightly. The biggest change is TCPP moving to the lower left quadrant due to revised statistics (which agrees with PM2.5 observations). This caused the movement of the ∑Cl-OPE for TSP as well, but as is pointed out in the original manuscript, ∑Cl-OPE is dominated by TCPP and typically follows its trend (or straddles categories based on influence of Cl-OPEs). Similarly, ∑Alkyl-OPE’s movement is due to the different quadrants of TBP and TEHP, which make it up, and changes in those concentrations in the 7/17/24 TSP sample. TCEP in PM_2.5_ was removed because less than half the date matched samples had detects.

**Figure 2:**
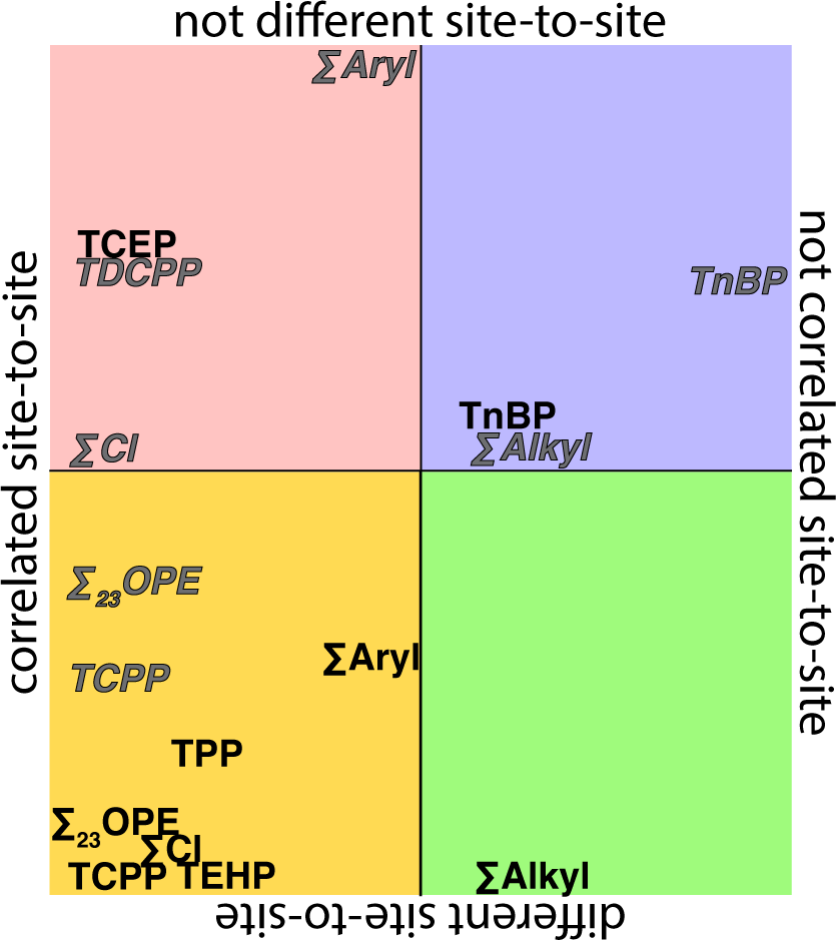
Comparison between sampling sites of Pearson correlation *p*-values and Student’s *t* test *p*-values (Table S3) for individual OPEs and summations for TSP samples (black) and PM_2.5_ samples (gray italicized).

For Figure 4, the change in TSP concentrations in one summer sample changed the placement of the summer TSP outlier (white) dot. The statistical significance of summer vs winter in TSP also changed, so letters were appropriately adjusted.

**Figure 4:**
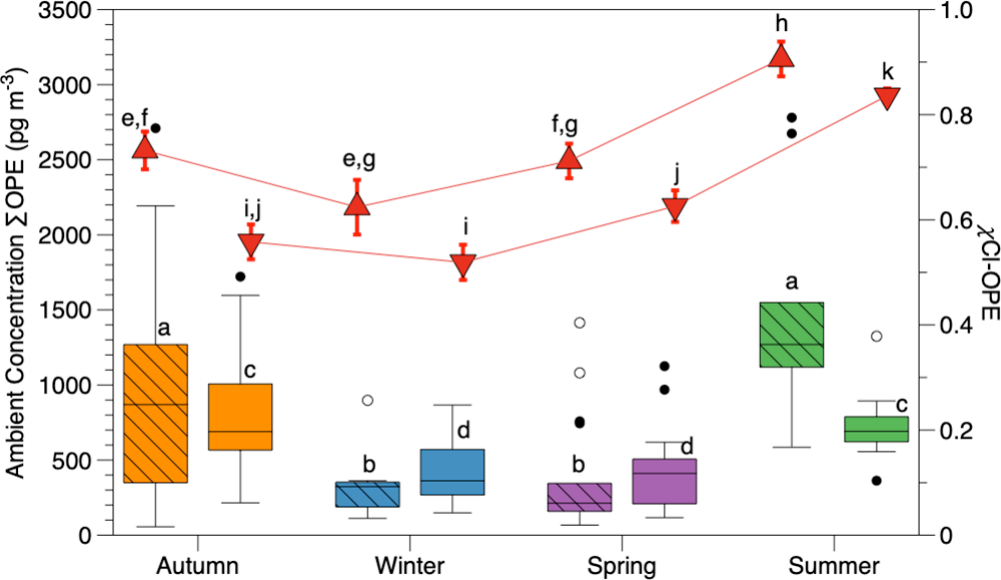
Box and whisker plots for ∑_23_OPE in PM_2.5_ (striped) and TSP (solid) samples from PC based on meteorological season as well as χCl for PM_2.5_ (red upward triangle) and TSP (red downward triangle), showing statistical differences between data sets.

## Supplementary Material



## Notes

(Table S1) Median, average, minimum and maximum, and detection frequency of all OPEs examined; (Table S2) average surrogate recoveries; (Tables S3–S8 and S10) Pearson correlation coefficients and *p*-values for correlation; (Tables S9, S11, and S12) *p*-values for two-tailed Student's t tests; (Table S13) list of compound names, abbreviations, method detection limits, and chemical structures for OPEs; (Figure S1) map of sampling locations; (Figure S2) results of NOAA HYSPLIT cluster analysis; (Figure S3) box and whisker plots; (Figure S4) NOAA HYSPLIT back trajectories (PDF)

