# Supplementary material for: Correction to “Spatioseasonal Variability and Correlations of Particle-Bound Organophosphate Esters in Providence, Rhode Island”
Source: ACS Omega. 2026 Jul 10;11(29):44539–42. doi: 10.1021/acsomega.6c05635 (PMC13425349; doi:10.1021/acsomega.6c05635)
Supplement: Supplementary file 1 [file ao6c05635_si_001.pdf]

Corrected Supporting Information for:

Spatioseasonal Variability and Correlations of Particle-Bound Organophosphate Esters in Providence, Rhode Island

*Annie Gathof, Leart Jahaj, Savannah Patalano, and Adelaide E. Clark**

Providence College, Department of Chemistry and Biochemistry, One Cunningham Square, Providence, RI, 02915.

*Corresponding Author email address: aclark6@providence.edu

Number of Pages: 23

Number of Tables: 13

Number of Figures: 4

Table S1. Median (Med), average (Avg), minimum and maximum (Range), and detection frequency (DF) of all OPEs examined in this study by size fraction and sampling site. <MDL indicates that more than half of the detected ambient concentrations were less than the method detection limits while N.D. indicates compound was not detected.

	Providence College								***Port of Providence***							
	**$PM_{2.5}$ (n = 63)**				**TSP (n = 66)**				**$PM_{2.5}$ (n = 26)**				**TSP (n = 23)**			
	**Med**	**Avg**	**Range**	**DF**	**Med**	**Avg**	**Range**	**DF**	**Med**	**Avg**	**Range**	**DF**	**Med**	**Avg**	**Range**	**DF**
TCEP	13	22	<MDL - 170	70	13	17	<MDL - 65	82	19	20	<MDL - 64	69	19	20	<MDL - 49	91
TCPP	353	488	9.2 - 3440	97	320	411	56 - 1766	100	422	615	105 - 3390	100	273	464	156 - 1450	100
TDCPP	154	352	<MDL - 1540	100	39	52	<MDL - 142	86	275	464	<MDL - 2400	92	<MDL		<MDL - 55	78
**∑Cl-OPE**	528	842	16 - 5160	100	388	469	73 - 1930	100	698	1060	116 - 5860	100	285	501	191 - 1480	100
TnBP	45	84	<MDL - 462	90	39	58	<MDL - 322	95	57	97	<MDL - 434	88	41	58	3.5 - 211	91
TEHP	<MDL		<MDL - 174	79	60	78	<MDL - 242	98	<MDL		<MDL - 56	96	220	198	54 - 327	100
**∑Alkyl-OPE**	53	89	<MDL - 568	98	124	131	<MDL - 416	100	75	110	<MDL - 455	100	254	251	68 - 379	100
TPP	<MDL		<MDL - 400	95	17	26	<MDL - 139	92	<MDL		<MDL - 114	77	21	29	<MDL - 95	91
EHDPP	<MDL		<MDL - 185	97	20	25	<MDL - 218	97	<MDL		<MDL - 84	92	<MDL		<MDL - 48	91
2IPPDPP	<MDL		<MDL - 165	59	<MDL		<MDL - 27	59	<MDL		<MDL - 28	59	<MDL			96
3IPPDPP	<MDL		<MDL - 34	22	<MDL			12	7.2	7.2	7.2	4	<MDL			9
4IPPDPP	<MDL		<MDL - 37	13	<MDL		<MDL - 118	35	14	15	<MDL - 31	31	<MDL		<MDL – 71	43
TOTP	9.4	30	<MDL - 305	48	13	16	<MDL - 56	24	<MDL		<MDL - 46	27	<MDL		<MDL - 47	43
TMTP	14	15	<MDL - 37	17	21	25	<MDL - 91	64	<MDL			8	12	12	<MDL - 16	30

	Providence College								*Port of Providence*							
	**$PM_{2.5}$ (n = 63)**				**TSP (n = 66)**				**$PM_{2.5}$ (n = 26)**				**TSP (n = 23)**			
	**Med**	**Avg**	**Range**	**DF**	**Med**	**Avg**	**Range**	**DF**	**Med**	**Avg**	**Range**	**DF**	**Med**	**Avg**	**Range**	**DF**
TPTP	<MDL			6	13	12	<MDL - 31	26	<MDL			4	10	11	<MDL - 17	17
2tBPDPP	<MDL			2	N.D.			0	N.D.			0	4.3	4.3	<MDL - 12	9
3tBPDPP	<MDL			22	<MDL		<MDL – 11	11	<MDL			8	<MDL			13
4tBPDPP	<MDL		<MDL - 53	83	<MDL		<MDL - 37	85	<MDL		<MDL - 24	65	<MDL		<MDL - 32	78
B2IPPPP	N.D.			0	<MDL		<MDL - 28	11	N.D.			0	N.D.			0
B3IPPPP	N.D.			0	N.D.			0	N.D.			0	<MDL			9
B4IPPPP	N.D.			0	N.D.			0	N.D.			0	N.D.			0
B2tBPPP	N.D.			0	N.D.			0	N.D.			0	N.D.			0
B3tBPPP	<MDL			21	<MDL		<MDL - 14	20	6.4	6.4	<MDL – 9.2	8	<MDL			4
B4tBPPP	<MDL		<MDL - 32	49	<MDL		<MDL - 18	53	<MDL			38	<MDL			48
24DIPPDPP	N.D.			0	N.D.			0	N.D.			0	N.D.			0
B24DIPPDPP	N.D.			0	N.D.			0	N.D.			0	N.D.			0
T2IPPP	<MDL			3	14	14	<MDL - 27	3	N.D.			0	<MDL			4
T3IPPP	N.D.			0	N.D.			0	N.D.			0	N.D.			0
T4IPPP	N.D.			0	N.D.			0	N.D.			0	<MDL			4
T34DMPP	N.D.			0	N.D.			0	N.D.			0	N.D.			0
T35DMPP	<MDL			2	<MDL			5	7.8	8.3	<MDL - 12	15	10	10	<MDL - 16	17
T3tBPP	N.D.			0	N.D.			0	N.D.			0	N.D.			0

	Providence College								*Port of Providence*							
	**$PM_{2.5}$ (n = 63)**				**TSP (n = 66)**				**$PM_{2.5}$ (n = 26)**				**TSP (n = 23)**			
	**Med**	**Avg**	**Range**	**DF**	**Med**	**Avg**	**Range**	**DF**	**Med**	**Avg**	**Range**	**DF**	**Med**	**Avg**	**Range**	**DF**
T4tBPP	<MDL			3	<MDL			3	N.D.			0	N.D.			0
**∑Aryl-OPE**	54	82	<MDL - 1080	100	64	95	<MDL - 530	100	51	60	8.5 - 276	100	78	84	46 - 163	100
**$\sum_{23}$OPE**	758	1010	56 - 6800	100	600	695	117 - 2590	100	889	1230	171 - 6270	100	689	836	374 - 1950	100

Table S2. Average surrogate recoveries by site and size fraction.

	**Providence College (PC)**		**Port of Providence (Port)**	
	$PM_{2.5}$ (n = 63)	*TSP (n = 66)*	*$PM_{2.5}$ (n = 26)*	*TSP (n = 23)*
d_{27}TnBP	69 ± 20%	79 ± 22%	75 ± 22%	85 ± 20%
d_{12}TCEP	71 ± 19%	84 ± 24%	70 ± 18%	77 ± 17%
d_{18}TCPP	73 ± 20%	84 ± 23%	81 ± 25%	87 ± 20%
d_{15}TDCPP	70 ± 19%	87 ± 25%	71 ± 19%	80 ± 16%
d_{15}TPP	66 ± 18%	77 ± 20%	66 ± 15%	69 ± 21%

Figure S1: Map of sampling locations in FriAir Net. The black pin indicates the location of Providence College (PC) sampling site; the white pin indicates the location of the Port of Providence sampling site.

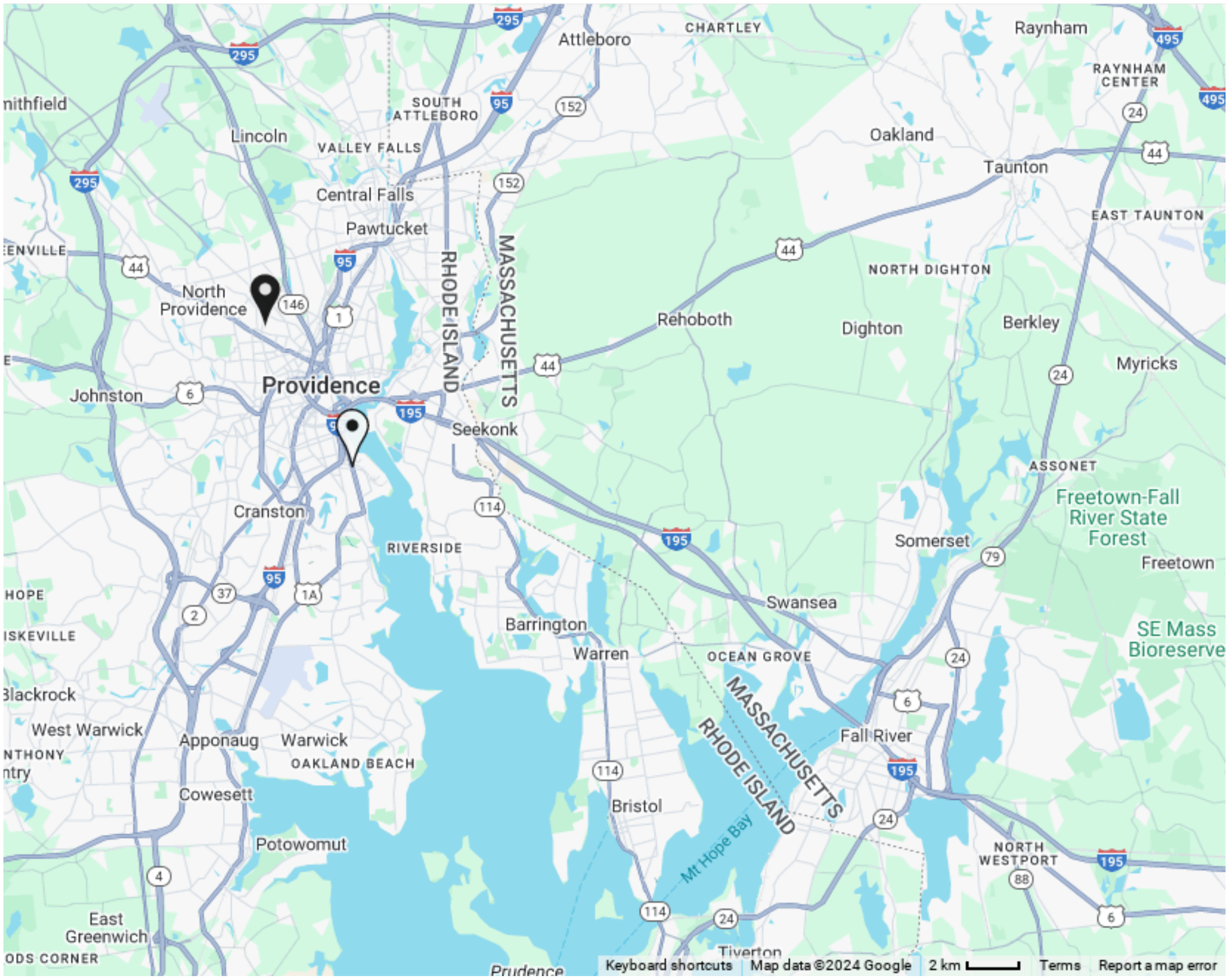

Table S3. Pearson correlation coefficients and p-values for correlation between sites (matched sample dates) and p-values for paired t-test of frequently detected OPEs (more than 50% >MDL) in TSP and $PM_{2.5}$ samples.

		TSP	$PM_{2.5}$			TSP	$PM_{2.5}$
TCEP	Pearson coeff.	**0.834**		TEHP	Pearson coeff.	**0.502**	
TSP: (n = 17)	p-value	**<0.001**		TSP: (n = 23)	p-value	**<0.05**	
	t-test p-value	0.291			t-test p-value	**<0.001**	
TCPP	Pearson coeff.	**0.908**	**0.810**	ΣCl	Pearson Coeff.	**0.919**	**0.854**
TSP: (n = 23)	p-value	**<0.001**	**<0.001**	TSP: (n = 23)	p-value	**<0.001**	**<0.001**
$PM_{2.5}$: (n = 25)	t-test p-value	**<0.001**	**<0.05**	$PM_{2.5}$: (n = 26)	t-test p-value	**<0.001**	0.054
TDCPP	Pearson coeff.		**0.877**	ΣAlkyl	Pearson Coeff.	0.383	0.371
$PM_{2.5}$: (n = 24)	p-value		**<0.001**	TSP: (n = 23)	p-value	0.072	0.068
	t-test p-value		0.281	$PM_{2.5}$: (n = 25)	t-test p-value	**<0.001**	0.053
TnBP	Pearson coeff.	0.438	0.251	ΣAryl	Pearson Coeff.	**0.429**	**0.416**
TSP: (n = 20)	p-value	0.061	0.284	TSP: (n = 23)	p-value	**<0.05**	**<0.05**
$PM_{2.5}$: (n = 22)	t-test p-value	0.090	0.269	$PM_{2.5}$: (n = 26)	t-test p-value	**<0.05**	0.549
TPP	Pearson coeff.	**0.568**		Σ_{23}OPE	Pearson Coeff.	**0.858**	**0.857**
TSP: (n = 20)	p-value	**<0.05**		TSP: (n = 23)	p-value	**<0.001**	**<0.001**
	t-test p-value	**<0.05**		$PM_{2.5}$: (n = 26)	t-test p-value	**<0.001**	**<0.05**

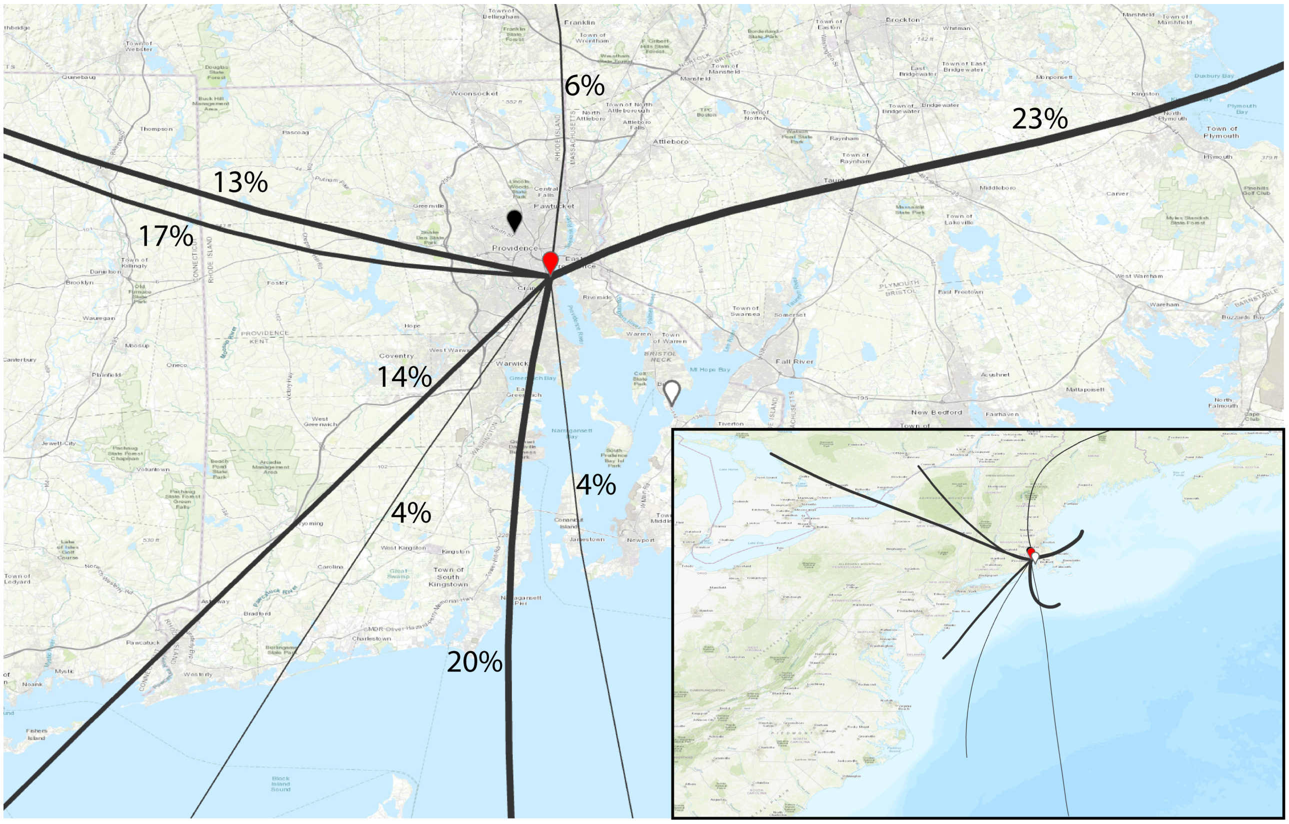

Figure S2. Results of NOAA HYSPLIT[1, 2] Cluster Analysis[3] (v. Jan 2010) with Providence College (black) and Port of Providence (red) highlighted. Each line represents a cluster of trajectories, with the relative width of the line corresponding to the percentage of trajectories it contains (also printed on the figure next to each line. Inset shows full extent of clustered trajectories.

Table S4. Pearson correlation coefficient and p-values for the association of frequently detected above MDL OPEs in PC TSP samples. Bold values indicate R>0.4 and p<0.05, while italics indicate R<0.4 and p<0.05.

		TCEP	TCPP	TDCPP	TPP	EHDPP	TEHP
TCPP	Pearson coeff.	**0.752**					
	p-value	**<0.001**[a,b,c]					
TDCPP	Pearson coeff.	**0.572**	**0.620**				
	p-value	**<0.001**[a,c]	**<0.001**[a,b,c]				
TPP	Pearson coeff.	*0.387*	**0.523**	0.268			
	p-value	*<0.01*[c]	**<0.001**[a,b,c]	0.063[a,b,c]			
EHDPP	Pearson coeff.	0.127	0.153	*0.309*	**0.429**		
	p-value	0.375[a,b,c]	0.227[a,c]	*0.023*[a,c]	**<0.005**[a,b,c]		
TEHP	Pearson coeff.	0.025	0.039	0.028	0.175	**0.442**	
	p-value	0.863[a]	0.757[a]	0.841[a]	0.198[a]	**<0.001**[a]	
TnBP	Pearson coeff.	**0.553**	**0.705**	**0.449**	**0.665**	**0.435**	0.127
	p-value	**<0.001**[a,c]	**<0.001**[a,c]	**<0.001**[a,b,c]	**<0.001**[a,b,c]	**<0.001**[a,b,c]	0.328[a]

[a] indicates that correlation has been previously observed in Great Lakes (2017)[4]

[b] indicates that correlation has been previously observed in Albany[5]

[c] indicates that correlation has been previously observed in Great Lakes (2012-2014)[6]

Table S5. Pearson correlation coefficients and p-values for the association of frequently detected above MDL OPEs in Port TSP samples. Bold values indicate R>0.4 and p<0.05.

		TCEP	TCPP	TEHP	TnBP
TCPP	Pearson coeff.	**0.682**			
	p-value	**<0.001**[a,c]			
TEHP	Pearson coeff.	-0.224	-0.061		
	p-value	0.330[b]	0.781[b]		
TnBP	Pearson coeff.	**0.843**	**0.722**	**-0.454**	
	p-value	**<0.001**[a]	**<0.001**[a]	**<0.05**[b]	
TPP	Pearson coeff.	**0.594**	**0.805**	-0.149	**0.754**
	p-value	**<0.01**	**<0.001**	0.519	**<0.001**

[a] indicates that correlation has been previously observed in Great Lakes (2017)[4]

[b] indicates that correlation has been previously observed in Great Lakes (2017),[4] but coefficient was positive.

[c] indicates that correlation has been previously observed in Albany[5]

Table S6. Pearson correlation coefficients and p-values for the association of frequently detected above MDL OPEs in PC $PM_{2.5}$ samples. Bold values indicate R>0.4 and p<0.05.

		TCEP	TCPP	TDCPP
TCPP	Pearson coeff.	**0.799**		
	p-value	**<0.001**[a]		
TDCPP	Pearson coeff.	**0.690**	**0.834**	
	p-value	**<0.001**	**<0.001**	
TnBP	Pearson coeff.	**0.741**	**0.654**	**0.502**
	p-value	**<0.001**[a]	**<0.001**[a]	**<0.001**[b]

[a] indicates correlation has been previously observed in Guangzhou[7]

[b] indicates correlation has been previously observed in Guangzhou,[7] but coefficient was negative

Table S7. Pearson correlation coefficients and p-values for the association of frequently detected above MDL OPEs in Port $PM_{2.5}$ samples. Bold values indicate R>0.4 and p<0.05.

		TCEP	TCPP	TDCPP
TCPP	Pearson coeff.	**0.694**		
	p-value	**<0.005**[a]		
TDCPP	Pearson coeff.	**0.560**	**0.849**	
	p-value	**<0.05**	**<0.001**	
TnBP	Pearson coeff.	**0.626**	**0.738**	0.371
	p-value	**<0.01**[a]	**<0.001**[a]	0.08[b]

[a] indicates correlation has been previously observed in Guangzhou[7]

[b] indicates correlation has been previously observed in Guangzhou,[7] but coefficient was negative

Table S8. Pearson correlation coefficients and p-values for correlation between size fractions (TSP v $PM_{2.5}$; matched sample dates) and p-values for paired t-test. Bold values indicate R>0.4 and p<0.05, while italics indicate R<0.4 and p<0.05.

		PC	Port			PC	Port
TnBP	Pearson Coeff.	**0.832**	0.322	ΣCl	Pearson Coeff.	**0.827**	**0.819**
	p-value	**<0.001**	0.260		p-value	**<0.001**	**<0.001**
	t-test p-value	**<0.05**[a]	0.275		t-test p-value	**<0.001**	0.138
TCEP	Pearson Coeff.	**0.581**	0.498	ΣAlkyl	Pearson Coeff.	**0.424**	-0.049
	p-value	**<0.001**	0.143		p-value	**<0.001**	0.847
	t-test p-value	0.186	0.339		t-test p-value	**<0.005**	**<0.001**
TCPP	Pearson Coeff.	**0.789**	**0.734**	ΣAryl	Pearson Coeff.	*0.354*	0.304
	p-value	**<0.001**	**<0.001**		p-value	*<0.005*	0.220
	t-test p-value	0.189	0.707		t-test p-value	0.526	0.190
TDCPP	Pearson Coeff.	**0.559**		Σ_{23}OPE	Pearson Coeff.	**0.772**	**0.773**
	p-value	**<0.001**			p-value	**<0.001**	**<0.001**
	t-test p-value	**<0.001**			t-test p-value	**<0.005**	0.394

[a] indicates that correlation has been previously observed in Houston[8]

Table S9. P-values for two-tailed student t-tests for comparing frequently detected OPEs, χCl, ∑Cl-, ∑Alkyl-, ∑Aryl-, and ∑OPE at PC in rain and non-rain samples. Bold values indicate $p<0.05$.

Compound	TSP	$PM_{2.5}$
TnBP	0.716	0.139
TCEP	0.746	0.335
TCPP	0.224	0.072
TDCPP	0.401	0.255
TPP	0.687	---
EHDPP	0.258	---
TEHP	**<0.005**	---
χCl	**<0.05**	0.342
∑Cl	0.262	0.101
∑Alkyl	0.186	0.224
∑Aryl	0.600	0.498
Σ_{23}OPE	0.778	0.088

Table S10. Pearson correlation coefficients and p-values for correlation between frequently detected OPEs, χCl, ∑Cl-, ∑Alkyl-, ∑Aryl-, and ∑OPE at PC and meteorological parameters. Bold values indicate R>0.4 and p<0.05, while italics indicate R<0.4 and p<0.05.

		Solar Radiation		Temperature		Barometric Pressure		Relative Humidity		Wind Speed	
		$PM_{2.5}$	TSP	$PM_{2.5}$	TSP	$PM_{2.5}$	TSP	$PM_{2.5}$	TSP	$PM_{2.5}$	TSP
TnBP	Pearson Coeff.	-0.108	-0.178	*0.339*	0.306	-0.054	-0.007	0.267	*0.373*	-0.257	**-0.454**
	p-value	0.506[b]	0.266	*<0.005*	0.051	0.739	0.965	0.095	*<0.05*	0.136	**<0.005**
TCEP	Pearson Coeff.	-0.153	0.030	0.343	**0.541**	0.027	-0.120	0.378	0.242	-0.280	*-0.388*
	p-value	0.446[b]	0.862	0.068	**<0.001**	0.894	0.493	0.056	0.161	0.162	*<0.05*
TCPP	Pearson Coeff.	-0.011	0.184	**0.674**	**0.741**	-0.254	-0.003	**0.525**	*0.324*	*-0.322*	**-0.591**
	p-value	0.945[a]	0.226	**<0.001[a,b,c]**	**<0.001**	0.096[a,c]	0.983	**<0.001**	*<0.05*	*<0.05*	**<0.001**
TDCPP	Pearson Coeff.	*0.304*	-0.090	**0.879**	-0.060	-0.199	0.243	**0.308**	0.083	*-0.334*	-0.222
	p-value	*<0.05[b]*	0.604	**<0.001[a,b]**	0.729	0.185[a]	0.153	**<0.05[a]**	0.630	*<0.05[b]*	0.222
TPP	Pearson Coeff.		-0.090		0.314		0.083		0.234		**-0.475**
	p-value		0.603		0.062		0.631		0.170		**<0.001**
EHDPP	Pearson Coeff.		-0.214		-0.061		0.067		0.075		*-0.378*
	p-value		0.168		0.793		0.670		0.631		*<0.05*
TEHP	Pearson Coeff.		-0.159		*-0.325*		0.287		-0.079		*-0.352*
	p-value		0.296		*<0.05*		0.056		0.604		*<0.05*
χCl	Pearson Coeff.	0.166	*0.296*	**0.636**	**0.523**	-0.166	0.010	**0.429**	0.196	-0.133	0.016
	p-value	0.269	*<0.05*	**<0.001**	**<0.001**	0.269	0.948	**<0.005**	0.198	0.407	0.924
ΣCl	Pearson Coeff.	0.135	0.170	**0.811**	**0.720**	-0.231	0.017	**0.475**	*0.340*	*-0.365*	**-0.596**
	p-value	0.371	0.263	**<0.001**	**<0.001**	0.123	0.911	**<0.001**	*<0.05*	*<0.05*	**<0.001**
ΣAlkyl	Pearson Coeff.	-0.076	-0.227	*0.396*	-0.051	-0.083	0.182	*0.300*	0.168	*-0.322*	**-0.528**
	p-value	0.619	0.134	*<0.01*	0.740	0.586	0.231	*<0.05*	0.269	*<0.05*	**<0.001**
ΣAryl	Pearson Coeff.	-0.166	-0.159	-0.152	0.158	0.142	0.094	0.154	0.192	**-0.446**	**-0.557**
	p-value	0.269	0.298	0.312	0.299	0.346	0.539	0.307	0.206	**<0.005**	**<0.001**

		Solar Radiation		Temperature		Barometric Pressure		Relative Humidity		Wind Speed	
		$PM_{2.5}$	TSP	$PM_{2.5}$	TSP	$PM_{2.5}$	TSP	$PM_{2.5}$	TSP	$PM_{2.5}$	TSP
Σ_{23}OPE	Pearson Coeff.	0.098	0.015	**0.777**	**0.511**	-0.205	0.082	**0.483**	*0.319*	**-0.408**	**-0.667**
	p-value	0.517	0.923	**<0.001**	**<0.001**	0.171	0.593	**<0.001**	*<0.05*	**<0.01**	**<0.001**

[a] indicates that correlation has been previously observed in Dalian[9]

[b] indicates that correlation has been previously observed in Guangzhou[7]

[c] indicates that correlation has been previously observed in South China[10]

Table S11. P-values for two-tailed student t-tests comparing frequently detected OPEs in $PM_{2.5}$ and TSP samples from PC for meteorological seasons. Bold values indicate at least $p<0.05$.

		$PM_{2.5}$			TSP		
		Autumn	Winter	Spring	Autumn	Winter	Spring
TnBP	Winter	**<0.05**			**<0.005**		
	Spring	**<0.05**	0.597		**<0.005**	0.844	
	Summer	0.180	0.059	0.149[a]	**<0.05**	0.545	0.498
TCEP	Winter	0.722			0.436		
	Spring	0.135	0.258		**<0.05**	**<0.05**	
	Summer	0.501	0.929	**<0.001**	0.293	**<0.05**	**<0.005**
TCPP	Winter	**<0.01**			**<0.01**		
	Spring	**<0.01**	0.911		**<0.05**	0.468	
	Summer	0.144	**<0.005**	**<0.005**[a]	0.091	**<0.005**	**<0.001**
TDCPP	Winter	**<0.01**			**<0.001**		
	Spring	0.058	0.108		**<0.001**	0.080	
	Summer	**<0.005**	**<0.001**	**<0.001**[a]	**<0.05**	**<0.001**	0.109
TPP	Winter				0.194		
	Spring				**<0.01**	0.516	
	Summer				**<0.05**	0.561	0.910
EHDPP	Winter				0.161		
	Spring				**<0.05**	0.184	
	Summer				**<0.05**	**<0.05**	0.060
TEHP	Winter				0.916		
	Spring				0.121	0.209	
	Summer				**<0.001**	**<0.05**	**<0.05**

[a]indicates that the same overall result was found for Port samples when compared, though p-value may have varied. Only spring and summer $PM_{2.5}$ samples were compared in this way.

Table S12. P-values for two-tailed student t-tests comparing summations and χCl in $PM_{2.5}$ and TSP samples from PC for meteorological seasons. Bold values indicate at least $p<0.05$.

		$PM_{2.5}$			TSP		
		Autumn	Winter	Spring	Autumn	Winter	Spring
χCl	Winter	0.125			0.444		
	Spring	0.681	0.168		0.137	**<0.05**	
	Summer	**<0.001**	**<0.005**	**<0.001**[a]	**<0.001**	**<0.001**	**<0.001**
ΣCl	Winter	**<0.005**			**<0.005**		
	Spring	**<0.05**	0.682		**<0.05**	0.452	
	Summer	**<0.05**	**<0.001**	**<0.001**[a]	0.105	**<0.001**	**<0.001**
ΣAlkyl	Winter	**<0.05**			0.074		
	Spring	**<0.05**	0.532		**<0.005**	0.363	
	Summer	0.191	0.105	0.060[a]	**<0.001**	0.074	0.154
ΣAryl	Winter	0.660			0.136		
	Spring	**<0.05**	0.342		**<0.01**	0.215	
	Summer	0.297	0.802	0.063[a]	**<0.005**	0.189	0.918
Σ_{23}OPE	Winter	**<0.01**			**<0.01**		
	Spring	**<0.01**	0.903		**<0.01**	0.960	
	Summer	0.066	**<0.001**	**<0.001**[a]	0.693	**<0.05**	**<0.01**

[a]indicates that the same overall result was found for Port samples when compared, though p-value may have varied. Only spring and summer $PM_{2.5}$ samples were compared in this way.

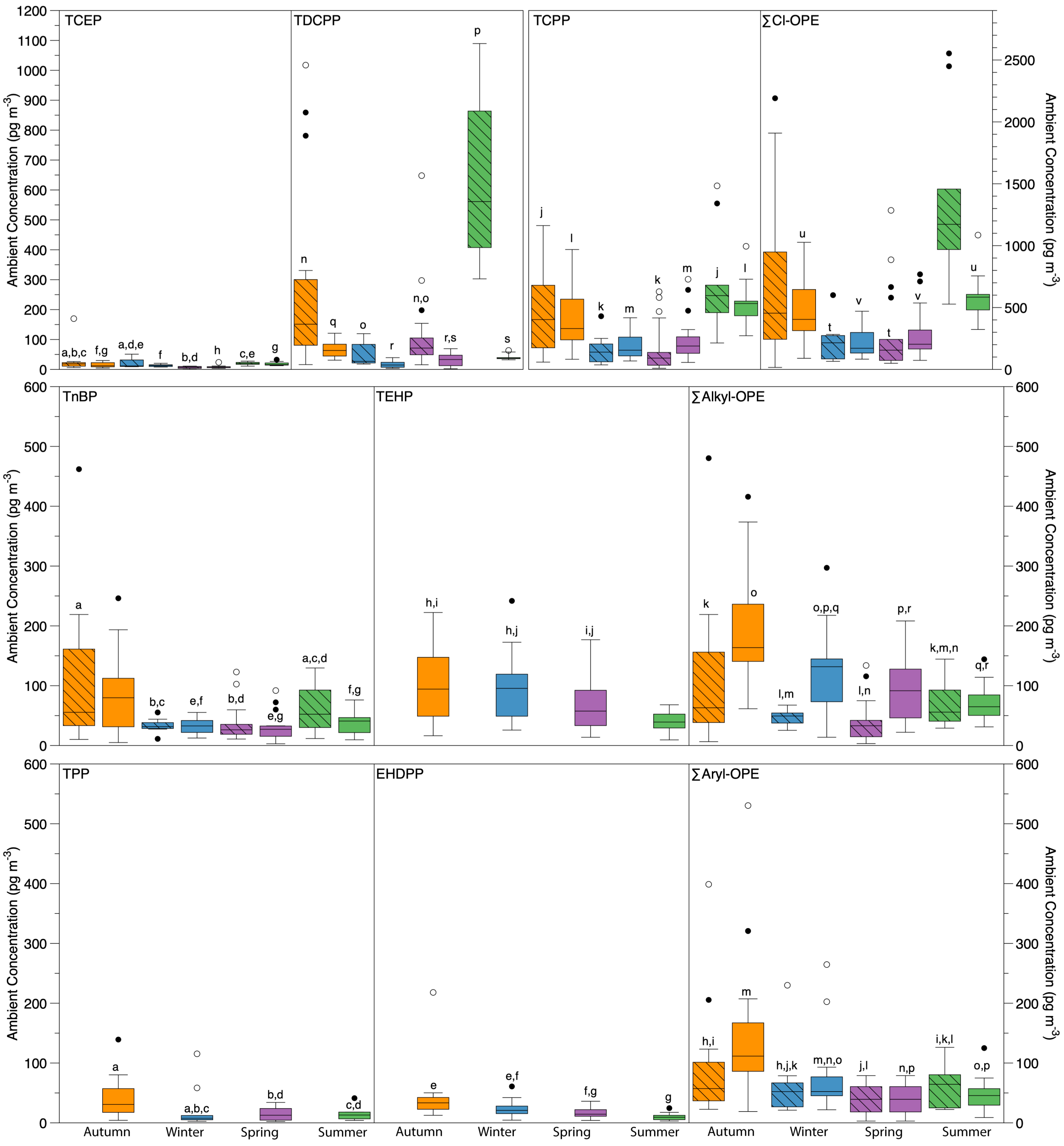

Figure S3. Box and whisker plots for TCEP, TDCPP, TCPP, and ∑Cl-OPE (top); TnBP, TEHP, and ∑Alkyl-OPE (middle); TPP, EHDPP, and ∑Aryl-OPE (bottom) in $PM_{2.5}$ (striped) and TSP (solid) samples from PC based on meteorological season (autumn: orange, winter: blue, spring: purple, summer: green), showing statistical differences between data sets.

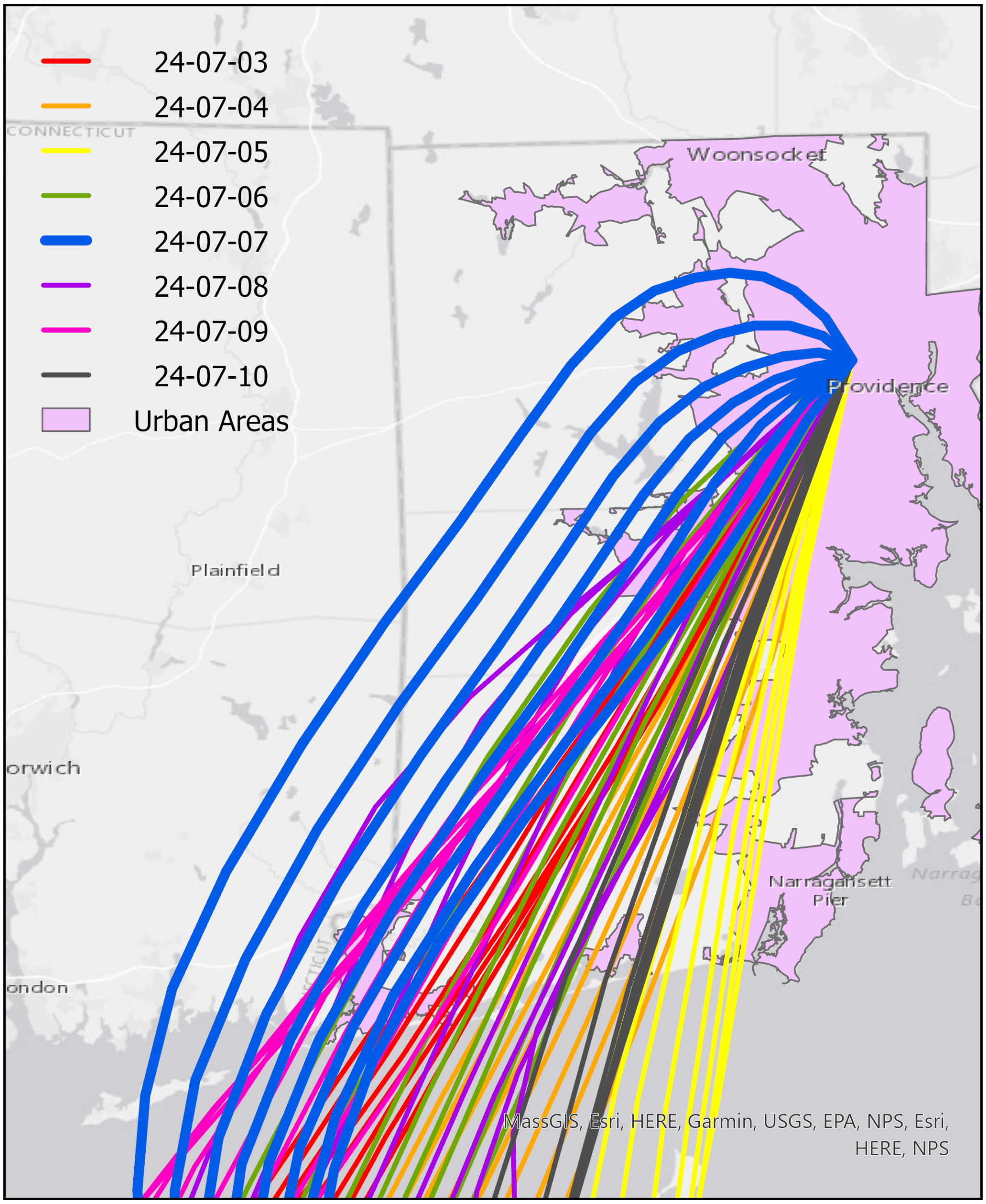

Figure S4. NOAA HYSPLIT[1, 2] back trajectories starting every hour for the 10 h night samples taken during the summer intensive. Each night is a different color, with the blue bold lines representing 24-07-07, which had the highest $\sum_{23}$OPE concentrations in PM2.5 and third highest in TSP. These trajectories moved through a different part of the region, passing through a different urban area (lilac) than other samples in the intensive, which may account for higher observed concentrations.

Table S13. List of compound names, abbreviations, method detection limits (MDLs) and chemical structure for OPEs included on target analyte list in this study. MDLs were previously published as part of methodology.[11]

Compound Name	Abbr.	MDL (ppb)	Chemical Structure
Tri-n-butyl phosphate	TBP	51.0	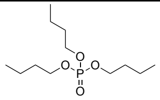
Tris(2-chloroethyl) phosphate	TCEP	14.2	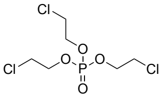
Tris[(2R)-1-chloro-2-propyl] phosphate	TCPP	41.1	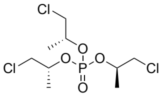
Tris(1, 3-dichloro-2-propyl) phosphate	TDCPP	39.7	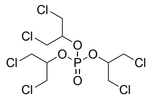
Triphenyl phosphate	TPP	53.4	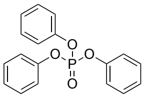
2-Ethylhexyl diphenyl phosphate	EHDPP	34.3	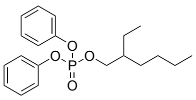
Tris(2-ethylhexyl) phosphate	TEHP	55.9	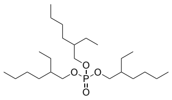
2-Isopropylphenyl diphenyl phosphate	2IPPDPP	26.7	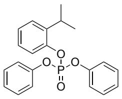
Tri-o-tolyl-phosphate	TOTP	27.4	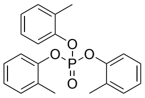
3-Isopropylphenyl diphenyl phosphate	3IPPDPP	24.0	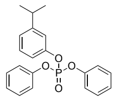
2-tert-Butylphenyl diphenyl phosphate	2tBPDPP	19.0	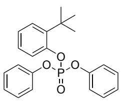

Compound Name	Abbr.	MDL (ppb)	Chemical Structure
Tri-m-tolyl-phosphate	TMTP	20.9	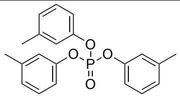
3-tert-butylphenyl diphenyl phosphate	3tBPDPP	19.2	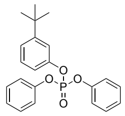
Bis(2-isopropylphenyl) phenyl phosphate	B2IPPPP	14.4	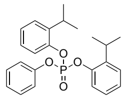
2,4-Diisopropylphenyl diphenyl phosphate	24DIPPDPP	18.2	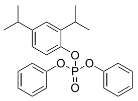
4-tert-Butylphenyl diphenyl phosphate	4tBPDPP	19.6	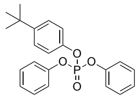
Tri-p-tolyl phosphate	TPTP	16.1	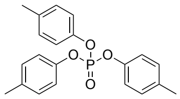
Bis(3-isopropylphenyl) phenyl phosphate	B3IPPPP	25.3	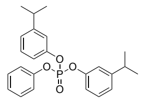
Tris(2-isopropylphenyl) phosphate	T2IPPP	19.4	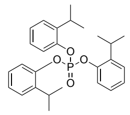
Bis(2-tert-butylphenyl) phenyl phosphate	B2tBPPP	17.7	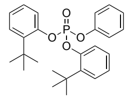
Tris(3,5-dimethylphenyl) phosphate	T35DMPP	21.9	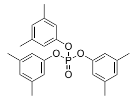
4-isopropylphenyl diphenyl phosphate	4IPPDPP	17.4	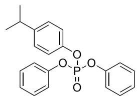
Bis(3-tert-butylphenyl) phenyl phosphate	B3tBPPP	22.0	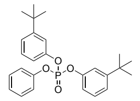

Compound Name	Abbr.	MDL (ppb)	Chemical Structure
Bis(4-isopropylphenyl) phenyl phosphate	B4IPPP	21.7	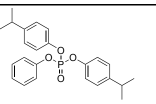
Tris(3-isopropylphenyl) phosphate	T3IPPP	24.5	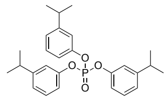
Bis(2,4-diisopropylphenyl) phenyl phosphate	B24DIPPPP	21.5	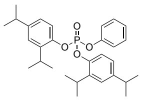
Tris(3,4-dimethylphenyl) phosphate	T34DMPP	17.6	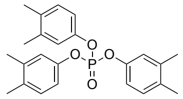
Tris(3-tert-butylphenyl) phosphate	T3tBPP	18.2	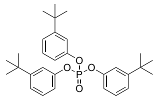
Bis(4-tert-butylphenyl) phenyl phosphate	B4tBPPP	22.9	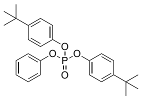
Tris(4-isopropylphenyl) phosphate	T4IPPP	20.2	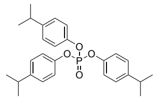
Tris(4-tert-butylphenyl) phosphate	T4tBPP	25.9	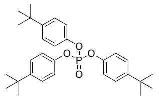

Supporting Information References

(1) Stein, A. F.; Draxler, R. R.; Rolph, G. D.; Stunder, B. J. B.; Cohen, M. D.; Ngan, F. NOAA's HYSPLIT Atmospheric Transport and Dispersion Modeling System. *Bullet Am Meteor Soc* **2015**, *96* (12), 2059-2077. DOI: 10.1175/BAMS-D-14-00110.1.

(2) Rolph, G.; Stein, A.; Stunder, B. Real-time Environmental Applications and Display sYstem: READY. *Environ Model Softw* **2017**, *95*, 210-228. DOI: 10.1016/j.envsoft.2017.06.025.

(3) Stunder, B. J. B. An Assessment of the Quality of Forecast Trajectories. *J Appl Meteor Climatol* 1996, 35 (8), 1319-1331. DOI: 10.1175/1520-0450(1996)035<1319:AAOTQO>2.0.CO;2.

(4) Wu, Y.; Venier, M.; Salamova, A. Spatioseasonal Variations and Partitioning Behavior of Organophosphate Esters in the Great Lakes Atmosphere. *Environ Sci Technol* **2020**, *54* (9), 5400-5408. DOI: 10.1021/acs.est.9b07755.

(5) Li, W.; Wang, Y.; Kannan, K. Occurrence, distribution and human exposure to 20 organophosphate esters in air, soil, pine needles, river water, and dust samples collected around an airport in New York state, United States. *Environ Int* **2019**, *131*, 105054. DOI: https://doi.org/10.1016/j.envint.2019.105054.

(6) Salamova, A.; Peverly, A. A.; Venier, M.; Hites, R. A. Spatial and Temporal Trends of Particle Phase Organophosphate Ester Concentrations in the Atmosphere of the Great Lakes. *Environ Sci Technol* **2016**, *50* (24), 13249-13255. DOI: 10.1021/acs.est.6b04789.

(7) Zeng, Y.; Chen, S. J.; Liang, Y. H.; Zhu, C. Y.; Liu, Z.; Guan, Y. F.; Ma, H. M.; Mai, B. X. Traditional and novel organophosphate esters (OPEs) in PM(2.5) of a megacity, southern China: Spatioseasonal variations, sources, and influencing factors. *Environ Pollut* **2021**, *284*, 117208. DOI: 10.1016/j.envpol.2021.117208.

(8) Clark, A. E.; Yoon, S.; Sheesley, R. J.; Usenko, S. Spatial and Temporal Distributions of Organophosphate Ester Concentrations from Atmospheric Particulate Matter Samples Collected across Houston, TX. *Environ Sci Technol* **2017**, *51* (8), 4239-4247. DOI: 10.1021/acs.est.7b00115.

(9) Wang, Y.; Bao, M.; Tan, F.; Qu, Z.; Zhang, Y.; Chen, J. Distribution of organophosphate esters between the gas phase and PM2.5 in urban Dalian, China. *Environ Pollut* **2020**, *259*, 113882. DOI: https://doi.org/10.1016/j.envpol.2019.113882.

(10) Zeng, Y.; Ding, N.; Wang, T.; Tian, M.; Fan, Y.; Wang, T.; Chen, S. J.; Mai, B. X. Organophosphate esters (OPEs) in fine particulate matter (PM(2.5)) in urban, e-waste, and background regions of South China. *J Hazard Mater* **2020**, *385*, 121583. DOI: 10.1016/j.jhazmat.2019.121583.

(11) Gathof, A.; Bonanno, T.; Rossicone, P.; Clark, A. E. Improved and Novel Methods for Investigating Organophosphate Esters in Particulate Matter. *Analytica*, 2024; Vol. 5, pp 471-480.
